# Global, regional, and national burdens of traumatic brain injury, spinal cord injury, and skull fracture and their attributable risk factors from 1990 to 2021: a systematic analysis of the global burden of disease study 2021

**DOI:** 10.3389/fpubh.2025.1622693

**Published:** 2025-08-20

**Authors:** Yikang Wang

**Affiliations:** Department of Neurosurgery, Shengjing Hospital of China Medical University, Shenyang, China

**Keywords:** traumatic brain injury, spinal cord injury, skull fracture, injury - head trauma, public health

## Abstract

**Background:**

Traumatic central nervous system (CNS) injuries—particularly traumatic brain injury (TBI), spinal cord injury (SCI), and skull fractures—represent a significant global health challenge. Previous estimates have lacked a comprehensive global analysis of these injuries and their associated risk factors. Herein, we aimed to examine the epidemiological patterns, temporal trends and risk factors of TBI, SCI, and skull fractures globally from 1990 to 2021.

**Methods:**

We extracted data from the Global Burden of Disease Study (GBD) 2021, including the prevalence, incidence, and years lived with disability (YLDs) of TBI, SCI, and skull fractures across 204 countries and territories from 1990 to 2021. Data were presented as both numerical counts and age-standardized rates (ASRs) per 100,000 population, with corresponding uncertainty intervals. To assess temporal trends in disease burden, we calculated the estimated annual percentage change (EAPC) with associated 95% confidence intervals.

**Results:**

Compared with 1990, the number of global incident cases in 2019 changed by 122.56, 121.29, and 97.49% for TBI, SCI, and skull fracture, respectively. During the 30-year study period, there was a downward trend in the ASR of prevalence, incidence and YLDs for TBI (EAPC = −0.68, −0.8 and −0.66, respectively), SCI (EAPC = −0.73, −0.81 and −1.01, respectively) and skull fracture (EAPC = −1.37, −1.15 and −1.38, respectively). Regions with higher sociodemographic indices had higher incidences, incidence rates, and YLDs for all three types of CNS injury. The burden of CNS injury varies notably among regions and nations. Eastern Europe, Central Europe, southern Latin America, Australasia, and high-income North America were the GBD regions with the highest burden of CNS injury, and the burdens of TBI, SCI, and skull fracture showed the most significant increasing trends in the Caribbean. Young-to-middle-aged men (15–39 years) bore the primary burden of TBI, SCI, and skull fractures. Falls were the leading specific risk factor for all three types of CNS injury, followed by motor vehicle road injuries. The global burden of TBI, SCI, and skull fractures is projected to decline through 2040, both in terms of absolute case counts and age-standardized incidence rates.

**Conclusion:**

The global, regional, and national burdens of TBI, SCI, and skull fractures—reflected by their prevalence, incidence, and YLDs—exhibit significant disparities. Our findings can inform policymakers in formulating future strategies for managing traumatic CNS injuries, with priority given to targeted preventive measures against risk factors to mitigate the burden of these life-threatening and disabling CNS conditions.

## Introduction

As a pressing global public health concern, central nervous system (CNS) injury profoundly impairs the quality of life of a substantial proportion of patients worldwide. Given the inadequate or limited efficacy of current therapeutic approaches, such injuries often result in fatal outcomes. Traumatic brain injury (TBI) is defined as damage to the brain induced by external mechanical force, which triggers hypoperfusion and hypoxemia; these pathological processes precipitate secondary injuries and may lead to temporary or permanent impairments in cognitive or physical function (1, 2). Patients with traumatic spinal cord injury (SCI) commonly exhibit significant long-term functional impairment or physical disability, imposing a profound burden on families and society (3). Skull fractures are a prevalent traumatic condition in neurosurgical practice, with most patients requiring timely surgical intervention (4). Amidst rapid global social development, the burden of these CNS injuries is steadily escalating, with a notable shift toward children and younger age groups. Accordingly, gaining a comprehensive understanding of their burden is of critical importance.

Globally, over 50 million individuals sustain TBI annually, with marked regional and national disparities in TBI burden. In the United States, TBI causes 50,000 deaths yearly, results in long-term impairments in 70,000 individuals, and affects 5.3 million people living with TBI-related disabilities (5, 6). In North Africa and the Middle East, mortality rates for TBI and SCI are significantly higher than in other regions, potentially linked to localized armed conflicts. In developing countries, the rising incidence of these injuries has coincided with increased automobile ownership (7). According to prior research, the incidence of TBI and SCI is projected to rise due to global population aging and inadequate road safety measures (8). Treatment and rehabilitation for TBI and SCI incur substantial costs, particularly for patients of low socioeconomic status (9). Lifetime costs for young patients with severe traumatic high cervical spinal cord injury may reach as high as $4.5 million, excluding indirect costs (10). In terms of functional outcomes, injuries such as skull fractures typically impose only short-term economic losses and family burdens. In contrast, cognitive impairments and neurological dysfunction from TBI, or quadriplegia and paraplegia resulting from high cervical SCI above the C5 level, may lead to lifelong deficits for patients (11). The poor outcomes associated with these injuries impose profound emotional and socioeconomic burdens on families and society. Consequently, beyond prioritizing injury prevention strategies, the healthcare system must anticipate the growing care burden for TBI and SCI patients to deliver a more robust healthcare response.

Amid substantial advances in the prevention and management of CNS injuries, the incidence and mortality rates of TBI, SCI, and skull fractures have declined steadily worldwide since the late 20th century (12). However, persistent challenges—including inadequate road infrastructure and limited medical facility coverage—have sustained high incidence rates in some countries/territories over time. Similarly, recent studies indicate that enduring disparities, such as regional inequities and socioeconomic gaps, issues associated with rapid urbanization, and fall risk closely linked to population aging, drive survival rate differences among patients with TBI and SCI (13). Given that associations between the burden of each type of CNS injury and sociodemographic characteristics across national and regional levels remain poorly understood, this study seeks to systematically elucidate the global, regional, and national burdens of TBI, SCI, and skull fractures.

The Global Burden of Disease Study (GBD) 2021 offers a unique opportunity to characterize temporal trends in the burden of TBI, SCI, and skull fractures over the past three decades. Leveraging the GBD dataset, this study provides a comprehensive analysis of the prevalence, incidence, and years lived with disability (YLDs) for these CNS injuries (14). Compared with previous GBD studies, this study offers unique strengths: it provides a detailed analysis of risk factors stratified by age and sex, conducts a combined analysis of three common CNS injuries in neurosurgical practice, analyzes the annual trends in the contribution ratio of risk factors—particularly falls due to aging and road traffic injuries caused by urbanization, and projects future trends in disease burden through 2040. Through this rigorous analysis, this study aims to comprehensively elucidate the burden of TBI, SCI, and skull fractures across national and regional levels from 1990 to 2021, aiming to inform healthcare decision-making and guide targeted prevention and treatment strategies.

## Methods

### Data acquisition

In this study, data from GBD 2021 were analyzed. This database employs the latest epidemiological data and standardized methodologies to conduct in-depth analyses of health losses associated with 369 diseases, injuries, and conditions, alongside 88 risk factors, across 204 countries and regions—thereby systematically evaluating global health status and disease burden. This study synthesized epidemiological data on CNS injury-related conditions, including TBI, SCI, and skull fractures. In the GBD 2021 study, data extraction utilized tools such as the GBD Results Tool and the Global Health Data Exchange platform, employing a standardized approach that integrates data from multiple sources (e.g., population-based registries, epidemiological surveys, and published studies) followed by systematic validation and harmonization to ensure data consistency^1^. More details about GBD 2021 have been presented in previous publications (14). The definitions of TBI, SCI and skull fracture are based on the International Classification of Diseases, 10th Revision standards.

As this study utilized publicly available aggregated data, no patient involvement occurred in the formulation of research questions, the determination of outcome measures, or study design and implementation. The University of Washington Institutional Review Board waived the informed consent requirement for access to GBD data (14). The data used in this study are publicly available and fully anonymized, thus ethical review is not required.

### Burden description

Our data analysis commenced with an exploration of the dataset’s structure by computing counts and rates for variables including prevalence, incidence, and YLDs of CNS injuries at global, regional, and national levels. We then analyzed metric variations from 1990 to 2019 across 204 countries and regions. When an injury cause resulted in multiple injury types, the most severe injury was selected using a mixed-effects regression model that accounts for age, sex, and uninjured status to estimate disability weights with national and individual random effects. Given that SCI is associated with greater disability than TBI and skull fractures, SCI was prioritized when all three injuries occurred due to the same cause.

Incidence and prevalence rates were calculated using the DisMod-MR 2.1 model, a Bayesian meta-regression framework for GBD modeling that integrates location, year, age, and sex data to estimate these metrics. DisMod-MR 2.1 incorporates diverse data types (such as surveillance studies, literature reviews, hospital discharge records, and emergency department data), quantifies uncertainties, integrates techniques such as meta-regression to construct rigorous epidemiological models, ultimately generating estimates of disease burden with spatiotemporal comparability, and supports rigorous epidemiological modeling (14, 15).

Incidence rates for cause-nature combinations were converted to prevalence rates using the differential equation solver integrated in DisMod-MR 2.1. YLDs were calculated using GBD disability weights, which are scored on a 0–1 scale (0 indicating complete health and 1 indicating death). Disability weights were determined via population and internet surveys, based on descriptions of disability status. For short-term disability, YLDs were computed by multiplying prevalence by GBD disability weights. For long-term disability, YLDs were calculated using a comparable approach, with adjustments for comorbidities; the cutoff for defining long-term disability was 1 year. Output variables included prevalence, incidence, and YLDs for each cause-nature combination, stratified by location, year, age, and sex. Finally, these metrics for TBI, SCI, and skull fractures were aggregated across all causes to compute overall values for each injury type separately (14, 15).

The sociodemographic index (SDI) is a composite metric developed by GBD researchers to quantify the development level of countries or regions. It is derived by integrating three components: the total fertility rate among individuals younger than 25 years, mean educational attainment among those aged 15 years and older, and lag-distributed per capita income, resulting in an index ranging from 0 to 1. A higher SDI value indicates a greater level of socioeconomic development. In this study, 204 countries and territories were categorized into five SDI strata—as defined in GBD 2021—including low SDI, low-middle SDI, middle SDI, middle-high SDI, and high SDI. This classification was used to examine associations between socioeconomic development and the burden of TBI, SCI, and skull fractures.

### Statistical analysis

In this study, age-standardized rates (ASRs) were utilized, including age-standardized prevalence rates (ASPR), age-standardized incidence rates (ASIR), and age-standardized YLD rates (ASYR) per 100,000 population. In GBD studies, low-SDI regions face challenges with data quality and underreporting due to weak surveillance systems, inefficient reporting mechanisms, and limited diagnostic capacity. To address these issues, GBD employs model-based imputation methods (e.g., multiple imputation, expectation–maximization, spatio-temporal Gaussian regression), which integrate partial data with covariates to estimate missing values. These approaches quantify uncertainty, ensuring robust disease burden analyses despite incomplete data. Further details are available in Supplementary material 1.

The estimated annual percentage change (EAPC) was calculated using Joinpoint regression, a statistical method that models temporal trends as piecewise linear functions on a logarithmic scale. The underlying model is formulated as: *ln(Yₜ) = α + βₖ(t - t₀) + εₜ*, where Yₜ denotes the ASR in year t, α represents the intercept, βₖ is the slope coefficient for the k-th segment, t₀ is the reference year, and εₜ signifies the random error term. This method employs permutation tests to iteratively detect significant joinpoints (i.e., points where the slope βₖ changes significantly) and determine the optimal number of trend segments. For each identified segment, EAPC is calculated as *(e^βₖ - 1) × 100*, quantifying the average annual percentage change. The overall EAPC integrates trends across all segments, with statistical significance assessed using *p*-values (*p* < 0.05 indicating significance). Ninety-five percent confidence intervals (95% CIs) for EAPC were computed. ASR trends upward if both EAPC and its 95% CI lower bound are positive; downward if both EAPC and its 95% CI upper bound are negative; otherwise, it is stable. EAPC effectively characterizes monotonic or non-monotonic temporal trends, thus enabling nuanced trend interpretation.

Age represents a major confounding factor in epidemiological comparisons: diseases often exhibit age-dependent patterns (e.g., higher incidence in older adults), and populations with divergent age compositions (e.g., a youthful population vs. an aging population) may show misleading differences in crude rates that reflect age structure rather than true variations in disease burden. ASRs were computed by applying weights derived from the GBD standard population to age-specific rates, a process designed to account for inherent variations in age distributions across populations. The formula is: *ASR = Σ* (age-specific rate for age group i × standard population proportion for age group i). The summation (Σ) across all age groups (i) integrates these weighted values, generating a single aggregate rate that is adjusted for age structure. This standardization facilitates cross-population or temporal comparisons by adjusting for confounding from age structure. Each of the three types of central nervous system injuries was modeled separately, with trend tests conducted (14, 15).

Percentage change (1990–2021) for prevalence/incidence was calculated as [(2021 cases − 1990 cases)/1990 cases] × 100. GBD 2021 geography comprises 204 countries/territories, aggregated into 21 regions and 5 SDI regions. ASPR, ASIR, ASYR, and EAPC were estimated annually by sex (1990–2021) across all geographical levels. World maps of 204 countries visualized these metrics for 1990 and 2021 (14, 15).

In this study, 95% uncertainty intervals (UIs) for prevalence, incidence, and YLDs were derived from GBD 2021 data. These 95% UIs were computed using Bayesian hierarchical models, which incorporate uncertainties from data variability, parameter estimation, and model structure. Monte Carlo simulations with 1,000 iterations generated posterior distributions, where UIs were defined as the 2.5th and 97.5th percentiles to capture 95% of the uncertainty range. A 95% UI that excluded zero was considered statistically significant. Statistical significance was additionally defined as a two-sided *p*-value < 0.05.

To predict future trends of CNS injuries, this study employed the Bayesian Age-Period-Cohort (BAPC) model, which incorporates integrated nested Laplace approximations for forecasting. The BAPC model is particularly valuable for projecting future rates based on historical data, making it a critical tool for public health planning and analysis. Further details are available in Supplementary material 2.

All statistical analyses and data visualizations were performed using R (version 4.4.2) and JD_GBDR (V2.37, Jingding Medical Technology Co., Ltd.). The R software package (version 4.2.3), GraphPad Prism (version 10.1.2) and JD_GBDR (V2.37, Jingding Medical Technology Co., Ltd.) was used for the drawing of the figures.

## Results

### Global level

Between 1990 and 2021, the global incidence of TBI experienced a 53.3% absolute increase, with the number of cases increasing from nearly 24.75 million to 37.93 million. Conversely, the ASPR decreased by 19.8%, from 536.7 to 448 cases per 100,000 individuals. The incidence rates increased by 22.6% in absolute terms, from nearly 17 million to 20.84 million. In contrast, the ASIR decreased by 25.3%, from 324.4 to 259 cases per 100,000 individuals. The ASYR for TBI in 2021 was 64.76 per 100,000 individuals, which decreased by 0.19 between 1990 and 2021. Furthermore, the ASIR from 1990 to 2021 experienced the sharpest decline with an EAPC of −0.8 (95% CI: −0.85 to −0.74). The ASPR has also decreased since 1990 (EAPC = −0.68 [95% CI, −0.72 to −0.63]), and the ASYR markedly declined from 1990 to 2021, with an EAPC of −0.66 (95% CI: −0.71 to −0.62) (Table 1; Supplementary Tables S1, S2).

**Table 1:** The incidence of TBI, SCI, and skull fracture in 1990 and 2021, and changes from 1990 to 2021 at the global level and different regions.

Characteristics	Number (95%UI)		Percentage change (95% UI)	ASIR (95% UI)		EAPC (95% CI)
	1990	2021		1990	2021	
Traumatic brain injury						
Global	17001269.3 (14859014.5–19464639.5)	20837465.8 (18128306.8–23839393.5)	22.56 (17.54 to 28.26)	324.4 (283.3–370.1)	259 (225.5–296.2)	−0.8 (−0.85 to −0.74)
Sex						
Male	11,580,571. (10236213.3–13126286.3)	13856451.3 (12201551.3–15600381.5)	19.65 (15.29 to 23.81)	436.8 (387.8–491.8)	347.2 (305.6–390.4)	−0.8 (−0.84 to −0.76)
Female	5420698.3 (4606004.3–6344007.2)	6981014.6 (5749070–8314257.1)	28.78 (21.03 to 37.56)	209.6 (177.7–245)	169.9 (140.7–201.8)	−0.77 (−0.87 to −0.68)
SDI						
High SDI	3524337.3 (2940299.6–4306393.5)	3549305.7 (2894216.1–4298864.8)	0.71 (−5.28 to 7.1)	399 (333.8–491.2)	305.3 (254.5–369.1)	−0.95 (−1 to −0.9)
High-middle SDI	4364788.1 (3795085.1–499,737)	4463101.3 (3843588.1–5142882.7)	2.25 (−2.67 to 7.58)	405.6 (353–465)	320.2 (277.4–370.2)	−0.95 (−1.04 to −0.86)
Middle SDI	5029617.9 (4434653.5–5693727.6)	6465389.6 (5624880.1–7383687.8)	28.55 (21.82 to 35.96)	300.8 (264.2–338)	258.6 (225.1–295)	−0.42 (−0.5 to −0.34)
Low-middle SDI	2865758.9 (2535090.1–3239563.6)	4205682.9 (3708320.4–4760273.1)	46.76 (41.24 to 52.36)	271 (239–305.9)	234.4 (205.5–266.6)	−0.63 (−0.78 to −0.47)
Low SDI	1195662.9 (1030974.6–1,426,572)	2133439.3 (1877861.8–2466372.7)	78.43 (70.23 to 86.04)	254.3 (223.5–294.8)	216.9 (191.5–246.7)	−0.46 (−0.73 to −0.2)
Regions						
Andean Latin America	104975.9 (92262.1–120575.8)	152863.8 (133549.8–173684.6)	45.62 (31.31 to 57.73)	275.5 (243.8–313.8)	229.7 (200.5–260.2)	−0.42 (−0.51 to −0.32)
Australasia	114283.2 (87639–152342.2)	139247.6 (105000.7–182131.8)	21.84 (14.07 to 31.72)	583 (440.3–789.6)	479 (355.1–652.1)	−0.58 (−0.66 to −0.5)
Caribbean	91005.7 (79930.2–102626.1)	144663.2 (125191.8–163811.4)	58.96 (50.16 to 68.76)	261.9 (229.2–295.3)	299.4 (259.9–338.8)	0.46 (−0.7 to 1.64)
Central Asia	257,707 (226936.4–292068.2)	269847.9 (237409.6–305727.4)	4.71 (2.62 to 6.77)	365.2 (324.1–412.4)	280.5 (246.8–318.6)	−1.05 (−1.24 to −0.86)
Central Europe	824837.5 (700734.1–951180.9)	601946.9 (506536.2–701295.2)	−27.02 (−30.47 to −23.77)	654.5 (557.5–755.7)	478.6 (405.1–560.6)	−1.22 (−1.29 to −1.15)
Central Latin America	730578.6 (635119.5–832662.9)	810782.8 (714279.9–915907.7)	10.98 (6.91 to 15.01)	451.3 (396.2–510.5)	318.3 (279.8–360.8)	−0.75 (−0.94 to −0.57)
Central Sub-Saharan Africa	107891.6 (95209.6–123223.6)	224834.5 (200748.2–253865.5)	108.39 (101.06 to 115.05)	204.4 (181.3–230.7)	183.1 (163.5–204.9)	−1.01 (−1.57 to −0.45)
East Asia	3061528.1 (2657683.9–3540501.4)	4313633.4 (3671116.3–4998871.5)	40.9 (30.76 to 52.13)	258.4 (224.6–294.9)	262.7 (224.7–304.7)	−0.16 (−0.36 to −0.04)
Eastern Europe	1597157.5 (1392360.6–1821788.6)	1115068.8 (963661–1274684.3)	−30.18 (−32.96 to −27.49)	698.3 (608.2–797.6)	522.4 (454.1–602.1)	−1.32 (−1.61 to −1.03)
Eastern Sub-Saharan Africa	535433.7 (430001.6–708467.3)	620976.5 (544555.5–720066.4)	15.98 (−3.86 to 38.13)	283.3 (233.3–361.1)	167.4 (148.2–190.5)	−1.48 (−1.98 to −0.97)
High-income Asia Pacific	662,604 (540910–824063.8)	446417.7 (357113.2–551238.6)	−32.63 (−37.53 to −27.34)	386.1 (313.8–485.2)	243.1 (191.5–312.8)	−1.71 (−1.81 to −1.6)
High-income North America	1041428.8 (858750.2–1285968.4)	1100572.5 (895390.5–1336998.5)	5.68 (−2.7 to 15.47)	368.1 (304.4–456.5)	269.2 (222.7–324.4)	−1.16 (−1.36 to −0.97)
North Africa and Middle East	1297462.9 (1148050–1471208.1)	2050666.5 (1801675.5–2,347,332)	58.05 (48.49 to 68.25)	384.7 (341.7–434.2)	333.4 (293.3–38)	0.01 (−0.18 to 0.17)
Oceania	11708.5 (10404–13074.2)	28898.7 (25663.6–32512.3)	146.82 (137.58 to 154.81)	198.1 (176.5–220.8)	229 (203.7–258.5)	0.13 (−0.41 to 0.68)
South Asia	2517619.6 (2194621.1–2883746.1)	4149216.6 (3543281–4823244.4)	64.81 (55.99 to 73.66)	267.7 (228.7–308.5)	243 (203.8–283.1)	−0.5 (−0.65 to −0.35)
Southeast Asia	1161654.8 (1032055.1–1316038.7)	1387481.8 (1217084.5–1564117.6)	19.44 (13.12 to 25.87)	258.4 (228.5–291.8)	201.1 (175.3–227.4)	−0.86 (−1.21 to −0.5)
Southern Latin America	191701.3 (146863.4–261616.4)	238177.7 (184977.5–321973.3)	24.24 (19.47 to 29.67)	382.3 (294.2–52)	362.7 (280.1–495.9)	−0.11 (−0.27 to −0.04)
Southern Sub-Saharan Africa	176838.9 (152479.5–202645.9)	203466.5 (176836.1–229678.9)	15.06 (11.16 to 19.59)	358 (309.2–409.7)	248.7 (217.7–279.6)	−1.35 (−1.48 to −1.22)
Tropical Latin America	661138.5 (568151.7–770714.3)	827052.5 (722080.9–948790.6)	25.1 (18.8 to 31.02)	440.6 (381.2–509.8)	351.3 (304.7–402.8)	−0.63 (−0.7 to −0.57)
Western Europe	1543939.4 (1256345.3–1898869.6)	1323852.6 (1043017.8–1667370.6)	−14.25 (−19.93 to −8.38)	405.3 (330–504.9)	300.3 (234.3–381)	−1.04 (−1.11 to −0.98)
Western Sub-Saharan Africa	309773.9 (274881.9–348015.1)	687797.2 (612197.8–774831.1)	122.03 (116.97 to 127.73)	172.3 (154.1–192.3)	162 (144.2–180.9)	−0.28 (−0.38 to −0.18)
Spinal cord injury						
Global	473666.3 (377726.3–598,764)	574502.3 (440218.9–757,445)	21.29 (11.16 to 33.65)	9.16 (7.28–11.68)	7.12 (5.48–9.36)	−0.81 (−0.94 to −0.69)
Sex						
Male	316978.7 (257439.4–391557.6)	369117.8 (292906.8–467308.7)	16.45 (7.87 to 26.09)	12.1 (9.75–14.95)	9.25 (7.34–11.72)	−0.81 (−0.91 to −0.72)
Female	156687.6 (119943.3–210696.7)	205384.5 (146802.6–292813.7)	31.08 (15.72 to 47.84)	6.17 (4.68–8.39)	4.94 (3.56–7.01)	−0.79 (−1.02 to −0.56)
SDI						
High SDI	123736.8 (97788.2–159244.6)	134,591 (98126.1–187691.6)	8.77 (−3.06 to 23.14)	13.5 (10.62–17.15)	10.4 (82–13.67)	−0.93 (−0.97 to −0.89)
High-middle SDI	108416.6 (85697.3–136933.3)	113558.3 (85504.5–155,268)	4.74 (−4.01 to 15.32)	10.1 (7.98–12.79)	7.96 (6.14–10.49)	−1.03 (−1.13 to −0.93)
Middle SDI	123337.7 (99330.3–156536.8)	152711.9 (116464–202186.1)	23.82 (7.73 to 41.03)	7.34 (5.83–9.31)	68 (4.66–89)	−0.1 (−0.36 to 0.16)
Low-middle SDI	71731.8 (56845–92699.7)	101,811 (79773.1–132364.4)	41.93 (26.26 to 55.27)	6.75 (5.26–8.83)	5.67 (4.38–7.51)	−0.82 (−1.15 to 0.49)
Low SDI	45939.1 (30226.7–72427.5)	71303.9 (51698.7–101058.9)	55.21 (36.26 to 76.56)	9.13 (6.23–148)	6.71 (4.95–9.23)	−0.75 (−1.64 to 0.15)
Regions						
Andean Latin America	3153.9 (2324.1–4413.9)	3370.4 (2670.3–4248.3)	6.87 (−25.43 to 38.74)	7.92 (5.94–10.79)	58 (41–6.43)	−0.9 (−1.23 to −0.58)
Australasia	3505.7 (2731.9–4461.4)	4,769 (3464.1–6695.4)	36.03 (19.08 to 57.41)	179 (13.36–21.76)	145 (10.51–18.8)	−0.55 (−0.62 to −0.49)
Caribbean	1911.3 (1560–2355.9)	3509.5 (2758.9–4514.7)	83.62 (65.78 to 109.21)	5.58 (4.49–6.94)	7.25 (5.71–9.27)	0.76 (−1.09 to 2.65)
Central Asia	5610.4 (4516.9–7051.8)	5985.2 (4809.5–7467.9)	6.68 (2.83 to 10.24)	7.98 (6.40–106)	6.22 (50–7.77)	−1.41 (−2.07 to −0.74)
Central Europe	18170.1 (13859.5–23760.9)	14138.1 (10176.9–19849.4)	−22.19 (−27.82 to −16.16)	14.2 (10.91–18.46)	10.8 (8.12–14.33)	−1.42 (−1.63 to −1.22)
Central Latin America	16802.7 (13359.7–21457.7)	17823.6 (14027.4–22716.3)	6.08 (−3.75 to 13.91)	10.4 (8.20–13.34)	6.98 (5.52–8.91)	−0.97 (−1.21 to −0.73)
Central Sub-Saharan Africa	2861.4 (2167.8–3843.2)	5,933 (4700.2–7,642)	107.35 (91.44 to 122.82)	5.34 (4.14–7)	4.67 (3.72–6)	−2.04 (−3.49 to −0.56)
East Asia	71649.1 (56609.5–91,187)	101621.6 (74344.8–139538.5)	41.83 (23.09 to 65.02)	66 (4.75–7.81)	6.12 (4.60–8.27)	−0.26 (−0.56 to 0.04)
Eastern Europe	35496.6 (27723–45628.8)	26392.7 (20167.4–35168.1)	−25.65 (−30.01 to −20.46)	15.4 (12.11–19.63)	12.2 (9.47–15.98)	−1.14 (−1.47 to −0.8)
Eastern Sub-Saharan Africa	29,273 (16217.7–51,831)	18,551 (13863.2–25919.7)	−36.63 (−56.94 to −5.75)	14.1 (8.30–24.58)	4.63 (3.55–6.36)	−3.12 (−4.54 to −1.68)
High-income Asia Pacific	24730.3 (19618.6–31638.7)	19157.6 (14175–26546.1)	−22.53 (−32.47 to −10.21)	13.8 (10.95–17.6)	8.57 (6.65–11.29)	−1.75 (−1.87 to −1.64)
High-income North America	39935.3 (31272–51314.4)	49938.8 (36906.6–68932.5)	25.05 (9.35 to 43.42)	13.6 (10.65–17.46)	11.2 (8.72–14.77)	−0.72 (−0.82 to −0.61)
North Africa and Middle East	31028.1 (24718.5–39925.2)	59433.7 (43804.6–80936.2)	91.55 (54.08 to 131.45)	9.25 (7.39–11.75)	9.56 (75–12.89)	1.57 (0.97 to 2.18)
Oceania	267.5 (214.2–342)	710.4 (563.1–932.5)	165.55 (150.95 to 182.28)	4.53 (3.60–5.84)	5.57 (4.33–7.43)	−0.08 (−1.03 to 0.89)
South Asia	66694.5 (51731.3–88547.9)	104040.6 (77913.7–142614.4)	56 (35.95 to 74.23)	7 (5.28–9.5)	67 (4.47–8.53)	−0.64 (−0.93 to −0.35)
Southeast Asia	29485.9 (23480.1–37749.5)	34341.2 (27164.7–43807.5)	16.47 (−1.72 to 34.9)	6.5 (5.14–8.26)	4.98 (3.91–6.4)	−0.82 (−1.62 to −0.01)
Southern Latin America	5574.4 (4437.5–6942.7)	7377.5 (5796.7–9352.8)	32.35 (27.06 to 37.99)	11.3 (8.98–148)	10.6 (8.40–13.45)	−0.17 (−0.28 to −0.07)
Southern Sub-Saharan Africa	3726.5 (2857.7–4951.2)	4340.9 (3356.6–5640.9)	16.49 (10.49 to 23.16)	7.47 (5.75–9.86)	5.3 (4.12–6.85)	−1.29 (−1.4 to −1.17)
Tropical Latin America	14795.6 (11391.5–19351.2)	19289.6 (14864.7–25414.2)	30.37 (22.84 to 38.08)	9.91 (7.63–132)	8.18 (6.33–10.73)	−0.25 (−0.38 to −0.12)
Western Europe	60735.6 (46471.7–81911.5)	55447.1 (38794.5–82255.9)	−8.71 (−20.31 to 3.73)	14.8 (11.52–19.56)	10.8 (7.96–14.83)	−1.12 (−1.18 to −1.06)
Western Sub-Saharan Africa	8258.4 (6491.6–10572.3)	18330.8 (14634.6–23499.8)	121.97 (108.26 to 134.48)	4.52 (3.57–5.86)	4.18 (3.32–5.41)	−0.31 (−0.63 to 0.01)
Skull fracture						
Global	5563809.2 (4415943.7–7124098.5)	5424294.6 (4212852.3–7235133.5)	−2.51 (−6.99 to 3.02)	98.6 (78.3–126.8)	70.1 (54.6–93)	−1.15 (−1.19 to −1.1)
Sex						
Male	4020070.1 (3228630.4–5112484.7)	3885356.1 (3068773.2–5094310.9)	−3.35 (−7.04 to 0.72)	140.5 (112.9–179.8)	99 (78.2–129.3)	−1.17 (−1.21 to −1.13)
Female	1543739.1 (1160908.7–2083658.7)	1538938.6 (1128561.1–2184970.8)	−0.31 (−7.82 to 8.16)	55.5 (41.8–75.2)	40.5 (30–57.1)	−1.09 (−1.16 to −1.01)
SDI						
High SDI	1352765.7 (1056790.2–1772467.3)	1130861.4 (855726.6–1548265.2)	−16.4 (−22.43 to −10.26)	162.2 (126.3–213.1)	118.1 (89.8–159.7)	−1.13 (−1.19 to −1.08)
High-middle SDI	1325122.4 (1026212.7–1712227.6)	1037573.4 (790363.3–1,419,981)	−21.7 (−26.43 to −16.02)	122.8 (95.5–159)	90.4 (69.5–122.3)	−1.18 (−1.25 to −1.1)
Middle SDI	1513311.6 (1207427.4–1963017.4)	1443823.4 (1108726.1–1943528.5)	−4.59 (−11.65 to 2.59)	79.6 (63.4–102.8)	61.2 (47.3–81.9)	−0.71 (−0.78 to −0.63)
Low-middle SDI	936728.2 (743559.9–1218169.9)	1,099,411 (865974.6–1450975.5)	17.37 (10.9 to 23.9)	71.9 (57.1–94)	54.9 (43.2–72.4)	−0.99 (−1.12 to −0.87)
Low SDI	429,281 (328616.4–603375.6)	707296.7 (550618.9–951252.8)	64.76 (54.57 to 74.72)	72.8 (55.9–103.1)	56 (43.8–74.6)	−0.71 (−0.97 to −0.46)
Regions						
Andean Latin America	3,954 (30758.3–51947.5)	46623.5 (35369–61533.3)	17.91 (−4.46 to 36.7)	90.6 (70.7–118.9)	68.8 (52.2–90.9)	−0.71 (−0.82 to −0.6)
Australasia	46820.5 (34165.1–63920.2)	51868.9 (36776.7–72,952)	10.78 (1.57 to 20.81)	244.5 (178.3–334.3)	199.7 (140.6–281.3)	−0.63 (−0.73 to −0.53)
Caribbean	29183.1 (22396.8–37553.2)	37406.9 (28811.5–47995.3)	28.18 (20.59 to 40.53)	76 (58.3–97.7)	82.2 (63.5–105.5)	0.21 (−0.51 to 0.93)
Central Asia	95286.3 (72152.7–124653.2)	90491.2 (67501.5–119695.3)	−5.03 (−7.53 to −2.13)	124.5 (94.5–162.4)	93.7 (69.7–124)	−1.15 (−1.34 to −0.96)
Central Europe	247278.8 (177275.6–335815.2)	139948.3 (97079–196257.1)	−43.4 (−46.76 to −39.99)	209.7 (150.8–283.8)	152.2 (107.2–213.5)	−1.19 (−1.26 to −1.11)
Central Latin America	256619.1 (196603.9–341840.2)	226983.2 (175163.8–302165.2)	−11.55 (−15.9 to −7.83)	134.6 (104.1–177.9)	91 (70.1–121.8)	−0.66 (−0.93 to −0.39)
Central Sub-Saharan Africa	37158.8 (28897.8–47824.5)	70,267 (56168.5–89703.8)	89.1 (79.08 to 101.01)	54.7 (43–70.6)	44.5 (35.5–56.9)	−1.55 (−2.32 to −0.78)
East Asia	774681.5 (591015.6–1051075.1)	717918.9 (517311.1–1020057.4)	−7.33 (−16.83 to 2.57)	60.9 (46.4–82.8)	54.3 (39.6–77.2)	−0.78 (−1.06 to −0.49)
Eastern Europe	455763.4 (348067.1–601577.3)	272051.9 (205358–367687.6)	−40.31 (−43.9 to −35.9)	213.8 (163.6–282.2)	151.6 (114.8–204.5)	−1.48 (−1.73 to −1.23)
Eastern Sub-Saharan Africa	204341.5 (137817–330,938)	217940.2 (172543.8–286474.1)	6.65 (−21.26 to 39.2)	88.6 (60.4–143.5)	44.8 (35.8–58.8)	−1.75 (−2.25 to −1.24)
High-income Asia Pacific	273,062 (210565.6–361222.1)	155,628 (115639.8–213526.1)	−43.01 (−48.19 to −38.4)	167.9 (130.3–222.7)	106.9 (79–146.3)	−1.64 (−1.75 to −1.52)
High-income North America	435243.6 (342053.2–564556.2)	390360.4 (301922.9–512043.2)	−10.31 (−16.96 to −2.71)	16 (125.8–205.8)	109.9 (86.9–141.6)	−1.42 (−1.64 to −1.2)
North Africa and Middle East	436128.7 (347494.2–559680.6)	602942.3 (469850.7–806,564)	38.25 (22.11 to 58.5)	110.8 (87.9–142.4)	93.5 (72.7–125.2)	−0.07 (−0.28 to 0.14)
Oceania	3352.4 (2651.2–4331.2)	7670.2 (5950.4–10178.5)	128.8 (113.59 to 143.61)	46.2 (36.5–59.5)	51.6 (39.9–68.6)	−0.02 (−0.49 to 0.46)
South Asia	780285.5 (601360.9–1069280.5)	963646.8 (728393.6–1,353,801)	23.5 (14.62 to 32.48)	65.3 (50.5–89.7)	50.8 (38.5–71.1)	−0.93 (−1.08 to −0.79)
Southeast Asia	363914.5 (289812.5–465971.3)	349810.7 (273154.7–452005.1)	−3.88 (−12.47 to 5.77)	70 (55.5–89.7)	50.4 (39.5–65.3)	−1.04 (−1.3 to −0.79)
Southern Latin America	83829.9 (63132–114450.2)	99174.9 (74852.2–136531.6)	18.3 (13.84 to 23.06)	165.8 (125.1–226.1)	156.3 (117.8–215.4)	−0.14 (−0.3 to 0.02)
Southern Sub-Saharan Africa	46965.6 (36293–59816.4)	49466.2 (38337–63376.4)	5.32 (1.04 to 9.87)	83.2 (63.9–107.6)	58.3 (45.3–74.7)	−1.29 (−1.41 to −1.17)
Tropical Latin America	215034.6 (164284.6–296610.8)	209308.9 (160673.9–283624.5)	−2.66 (−7.55 to 2.73)	127.3 (97.1–174.4)	95.1 (73.2–129.8)	−0.87 (−1.01 to −0.73)
Western Europe	626759.6 (467802.1–863657.3)	484981.3 (341985.1–708622.1)	−22.62 (−30.19 to −15.93)	179.6 (134.2–248.4)	136 (96.8–197.8)	−0.96 (−1.05 to −0.88)
Western Sub-Saharan Africa	112559.8 (90193.6–142469.6)	239804.8 (192203.4–307796.9)	113.05 (104.36 to 122.76)	48.2 (38.6–61.4)	42.1 (33.4–54.3)	−0.54 (−0.67 to −0.4)

TBI, traumatic brain injury; SCI, spinal cord injury; UI, uncertainty interval; CI, confidence interval; EAPC, estimated annual percentage change; ASIR, age-standardized incidence rates.

The number of cases of SCI worldwide also increased from approximately 10.82 million in 1990 to 15.4 million in 2021. The ASPR of SCI decreased from 222.7 per 100,000 individuals to 183.6 over the same time period (EAPC = −0.73 [95% CI, −0.77 to −0.69]). In 2021, the global incidence of SCI was 0.57 million, with an ASIR of 7.12 per 100,000 individuals, with a decrease in the ASIR (EAPC = −0.81 [95% CI, −0.94 to −0.69]) and an increase in the incidence of SCI (percentage change = 0.21) from 1990 to 2021. The ASYR of SCI also decreased significantly between 1990 and 2001, from 71.78 per 100,000 individuals in 1990 to 54.62 per 100,000 individuals in 2021, with an EAPC of −1.01 (95% CI: −1.04 to −0.95) (Table 1; Supplementary Tables S1, S2).

Globally, the number of cases of skull fracture reported in 2021 was 23.98 million, representing a 19.6% increase from 1990 (20.05 million). The ASPR of skull fracture decreased from 42.95 per 100,000 individuals in 1990 to 29.04 per 100,000 individuals in 2021. During this period of our study, the EAPC in the ASPR was −1.37 (95% CI: −1.41 to −1.33). The incidence of skull fracture was estimated to be 5.56 million in 1990 and 5.42 million in 2021. Although the absolute number of incidence cases remained stable, the ASIR decreased from 98.59 per 100,000 individuals in 1990 to 70.12 per 100,000 individuals in 2021. From 1990 to 2021, the EAPC for the ASIR of skull fracture was −1.15 (95% CI: −1.19 to −1.1) (Table 1; Supplementary Tables S1, S2).

### Regional level

**Figure 1:**
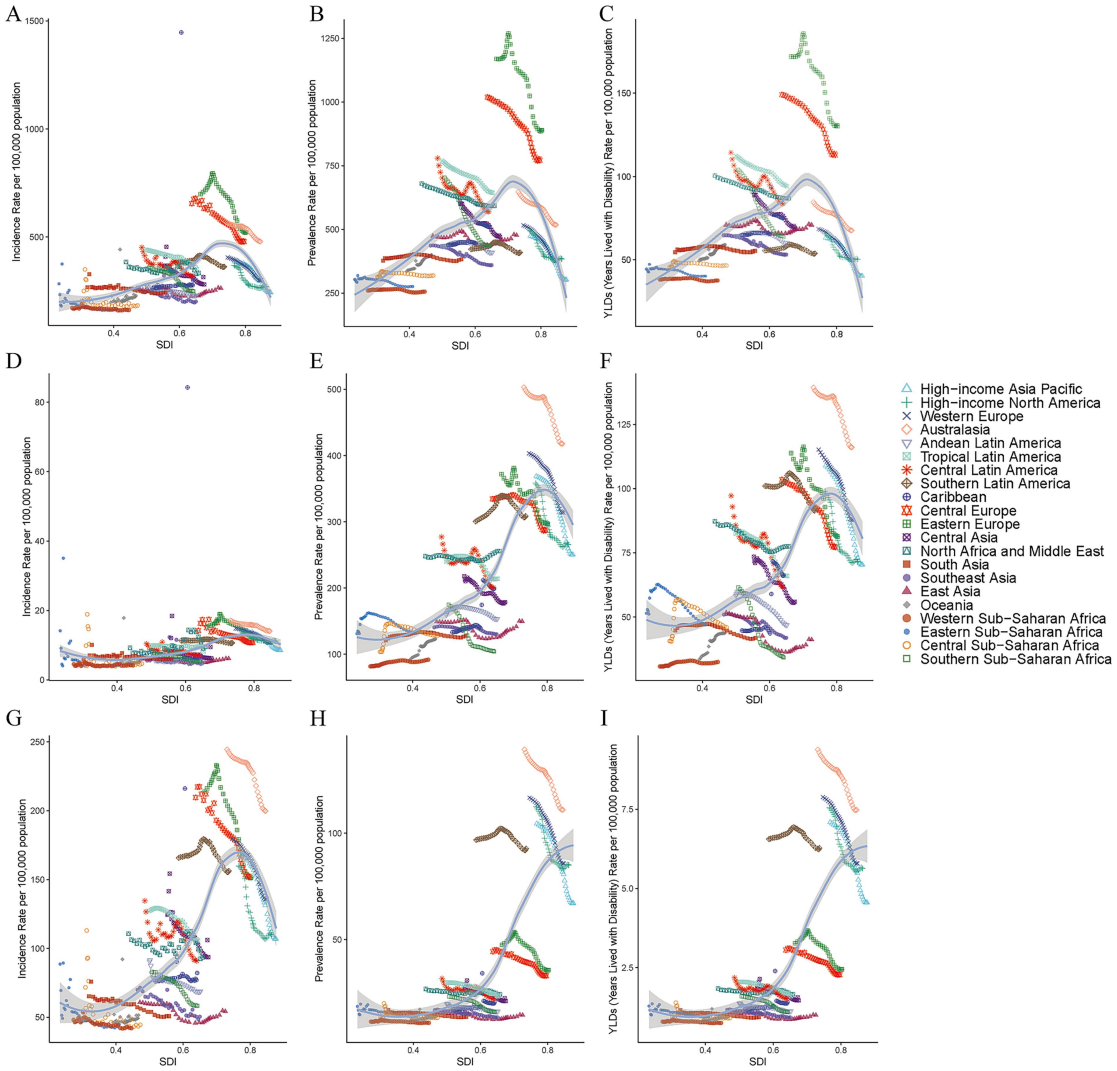
Age-standardized incidence rates of TBI **(A)**, SCI **(B)** and skull fracture **(C)** for 21 regions according to the SDI, 1990–2021. Age-standardized incidence rates of TBI **(D)**, SCI **(E)** and skull fracture **(F)** for 21 regions by the SDI, 1990–2021. Age-standardized years lived with disability rates of TBI **(G)**, SCI **(H)** and skull fracture **(I)** for 21 regions according to the SDI, 1990–2021. The expected values are shown as black lines on the basis of the SDI and disease rate. TBI, traumatic brain injury; SCI, spinal cord injury; SDI, sociodemographic index.

**Figure 2:**
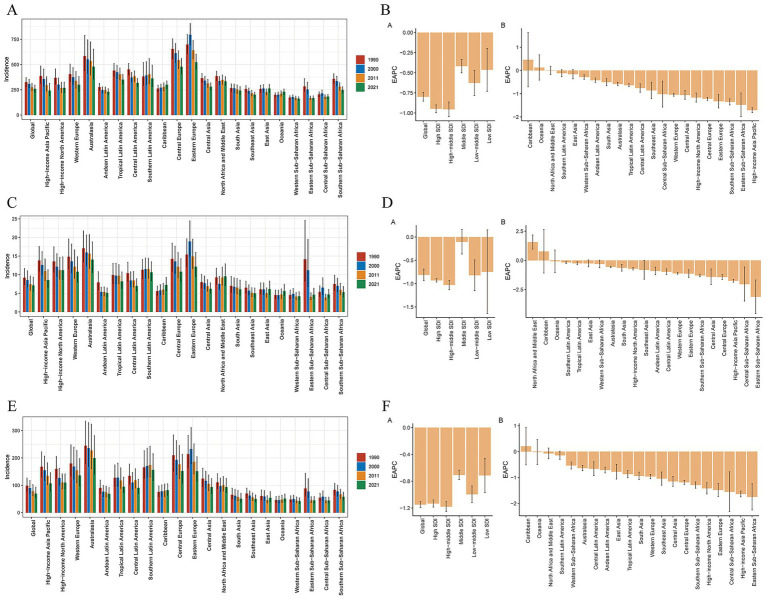
ASIRs in 2021 and their annual changes every decade from 1990 to 2021 for TBI, globally and by 21 GBD regions **(A)**, and the EAPCs in the ASIRs across 21 GBD regions from 1990 to 2021 **(B)**. ASIRs in 2021 and their annual changes every decade from 1990 to 2021 for the SCI, globally and by 21 GBD regions **(C)**, and the EAPCs of the ASIRs across 21 GBD regions, 1990–2021 **(D)**. ASIRs in 2021 and their annual changes every decade from 1990 to 2021 for skull fracture, globally and by 21 GBD regions **(E)**, and EAPCs in the ASIRs across 21 GBD regions, 1990–2021 **(F)**. ASIR, age-standardized incidence rate; EAPC, estimated annual percentage change; TBI, traumatic brain injury; SCI, spinal cord injury.

From 1990 to 2021, there were significant variations in the incidence of TBI across 21 regions. The highest ASPR in 2021 was observed in Eastern Europe at 888.9 per 100,000 individuals, whereas Western Sub-Saharan Africa recorded the lowest ASPR at 256.1 per 100,000 individuals. Southern Sub-Saharan Africa displayed the most substantial decline in its ASPR, with a relative change in the EAPC of −1.73 (95% CI: −1.82 − 1.64). In contrast, the Caribbean exhibited the greatest increase in the incidence of TBI, as evidenced by an EAPC of 0.71 (95% CI: 0.5–0.92) (Figure 1B; Supplementary Table S1; Supplementary Figures S1A,B). Among the 21 GBD regions, the highest ASYRs of TBI were found in Eastern Europe (130.3), Central Europe (112.9) and Tropical Latin America (94.1), whereas the highest ASIRs were recorded in Eastern Europe (522.4), Australasia (479) and Central Europe (478.6) (Table 1; Supplementary Table S2). In 2021, the lowest ASIR per 100,000 individuals was reported in Central Sub-Saharan Africa (183.1), whereas the lowest ASYR was reported in the high-income Asia Pacific region (126.2). From 1990 to 2021, the ASIR of TBI decreased across all 21 global regions except the Caribbean (EAPC = 0.46 [95% CI, −0.7 to 1.64]) and Oceania (EAPC = 0.13 [95% CI, −0.41 to 0.68]). Similarly, in terms of the ASYR, only the Caribbean (EAPC = 0.7 [95% CI, −0.46 to 0.94]) and Oceania (EAPC = 0.56 [95% CI, 0.5 to 0.62]) countries presented consistent upward trends. Additionally, in our study, the most rapid decrease in the ASYR of TBI was observed in southern Sub-Saharan Africa, with an EAPC of −1.76 (95% CI: −1.85 to −1.67), whereas the most significant decrease in the ASIR was reported in the high-income Asia Pacific region (EAPC = −1.71 [95% CI, −1.81 to −1.6]) (Figures 1A,C, 2A,B; Supplementary Figures S2A,B).

In 2021, studies conducted at various regional levels worldwide revealed that the ASPR of SCI was highest in Australasia (417.6), Western Europe (313.9) and southern Latin America (308.7), whereas the lowest was in western sub-Saharan Africa (91.8) (Supplementary Table S1, Supplementary Figure S1C,D). The ASIR for SCI was highest in Australasia (14.1), followed by Eastern Europe (12.2) and high-income North America (11.2). In contrast, western Sub-Saharan Africa presented the lowest ASIR, at 4.18 per 100,000 individuals in 2021 (Table 1). With respect to the ASYR of SCI in 21 geographical regions, the highest ASYR in 2021 was found in Australasia (116), southern Latin America (92.2) and Western Europe (87.7), and the lowest was in western Sub-Saharan Africa (32.5). During the period from 1990 to 2021, changes in the SCI burden varied across different world regions. The most notable reductions in the ASYR and ASPR were both observed in southern Sub-Saharan Africa, with an EAPC of −2.04 (95% CI: −2.27 to −1.81) for the ASYR and an EAPC of −1.81 (95% CI: −2.05 to −1.56) for the ASPR (Figures 1E,F). Moreover, it is noteworthy that from 1990 to 2021, the Caribbean experienced the most significant upward trend in ASPR (EAPC = 1.77 [95% CI, 1.4 to 2.14]) and ASYR (EAPC = 1.85 [95% CI, 1.38 to 2.31]) (Supplementary Tables S1, S2; Supplementary Figures S1C,D, S2C,D). North Africa and the Middle East presented the most significant upward trend in the ASIR, at an EAPC of 1.57 (95% CI: 0.97–2.18), whereas eastern Sub-Saharan Africa (EAPC = −3.12 [95% CI, −4.54 to −1.68]) presented the most pronounced decline in the ASIR of the SCI (Figures 1D, 2C,D).

Among the 21 geographical regions worldwide in 2021, the ASIR of skull fracture in young adults was highest in Australasia (199.7), followed by southern Latin America (156.3) and Central Europe (152.2), whereas it was lowest in Western Sub-Saharan Africa (42.1). In 2021, Australasia (110.97) had the highest ASPR of skull fracture, whereas the rates were lowest in western sub-Saharan Africa (10.91) (Table 1; Supplementary Table S1). From 1990 to 2021, the ASIR and ASPR of skull fracture worldwide tended to decrease, with the exception of the Caribbean, which presented the most significant increasing trends in the ASIR (EAPC = 0.21 [95% CI, −4.54 to −1.68]) and ASPR (EAPC = 0.76 [95% CI, 0.34 to 1.19]), and the largest decreases in the ASIR occurred in Eastern Sub-Saharan Africa (EAPC = −1.75 [95% CI, −2.25 to −1.24]). Moreover, the most notable decline in the ASIR was observed in high-income Asian Pacific region (EAPC = −1.62 [95% CI, −1.75 to −1.52]) (Figures 1G,H, 2E,F; Supplementary Figures S1E,F). Australasia had the highest ASYR (7.49) of skull fracture in 2021, whereas western Sub-Saharan Africa (0.75) had the lowest ASYR. Significant changes in the ASYR were particularly evident from 1990 to 2021. Notably, the Caribbean (EAPC = 0.71 [95% CI, 0.28 to 1.14]) experienced the most substantial relative increases, and the most predominant downward trend was observed in the high-income Asia Pacific region (EAPC = −1.63 [95% CI, −1.75 to −1.52]) (Supplementary Table S2; Supplementary Figures S2E,F; Figure 1I).

**Figure 3:**
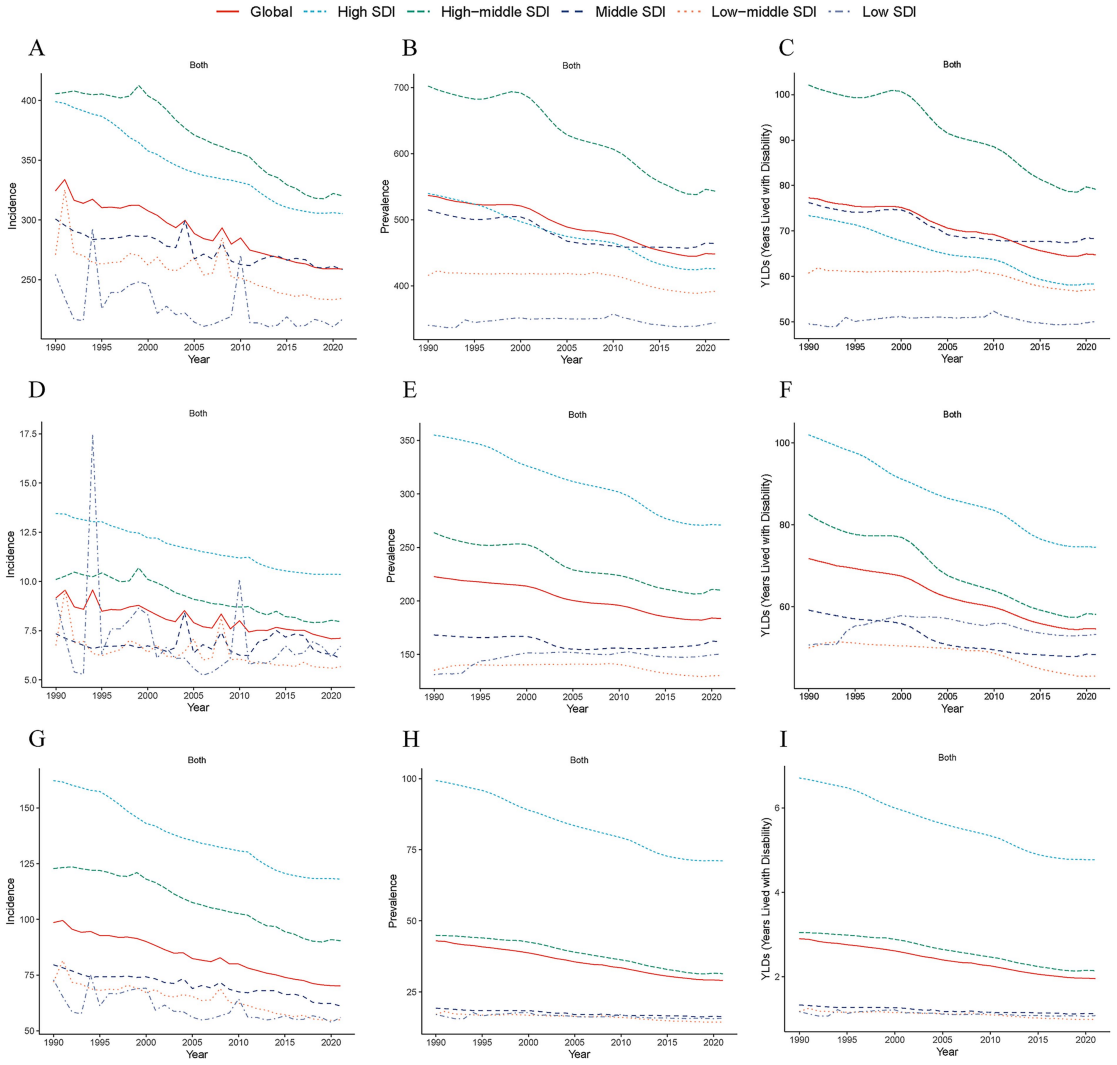
Temporal trends in age-standardized incidence rates of TBI **(A)**, SCI **(B)** and skull fracture **(C)** across 5 SDI regions (high-SDI, high–middle-SDI, middle-SDI, low–middle-SDI, and low-SDI categories) from 1990 to 2021. Temporal trends in age-standardized incidence rates of TBI **(D)**, SCI **(E)** and skull fracture **(F)** across 5 SDI regions from 1990 to 2021. Temporal trends in age-standardized years lived with disability rates of TBI **(G)**, SCI **(H)** and skull fracture **(I)** across 5 SDI regions from 1990 to 2021. TBI, traumatic brain injury; SCI, spinal cord injury; SDI, sociodemographic index.

The global burden of CNS injury exhibits significant regional differences closely related to SDI levels. Temporal trends in the ASPR, ASIR and ASYR of CNS injury revealed divergent patterns across SDI levels, and the changes in the burdens of TBI, SCI and skull fracture between 1990 and 2021 exhibited a declining trend. Specifically, the burden of TBI was the highest in high−middle-SDI regions, followed by high-SDI regions, indicating the need for increased attention to the TBI burden in this population (Figures 3A–C). Additionally, high-SDI regions bear the highest burden of SCI globally, followed by high−middle-SDI regions, with regional disparities in the burden of SCI (Figures 3D–F). In addition, this SDI region analysis suggests that high-SDI regions face a particularly high burden of skull fracture, impacting the prevalence rate, incidence rates and overall health-related quality of life among their populations. These patterns underscore the persistent global burden of CNS injury, particularly in regions with high–middle and high SDIs (Figures 3G–I).

### National level

From 1990 to 2021, the burdens of TBI, SCI and skull fracture markedly varied across 204 countries and territories (Supplementary Figure S5). At the national level, the highest ASPR of TBI was observed in Saudi Arabia (1218.2), Slovenia (974.6), and Afghanistan (968.9). In contrast, Madagascar (175.8) displayed the lowest ASPR (Supplementary Table S4; Supplementary Figure S3A). In 2021, the Syrian Arab Republic (816.1), Afghanistan (787.2), and Iraq (618.3) had the highest ASPR of SCI, and Malawi (57.4) exhibited the lowest ASPR (Supplementary Table S7; Supplementary Figure S3C). Among all the countries, New Zealand (126.9), Andorra (128.1), and Finland (114.9) presented the highest ASPR of skull fracture (Supplementary Table S11; Supplementary Figure S3E). For holistic appraisal of the observed trends, considering the ASPR of TBI, Burundi (EAPC = 2.42 [95% CI, 1.47 to 3.37]) reported the steepest increase, whereas Portugal (EAPC = −2.51 [95% CI, −2.63 to −2.38]) presented the greatest decrease (Supplementary Table S4; Supplementary Figure S3B). With respect to the ASPC of SCI, 81 countries experienced an increase, with Burundi’s EAPC of 6.92 (95% CI: 4.65 to 9.25) at the forefront. Moreover, Mozambique experienced the most substantial decline at an EAPC of −3.16 (95% CI: −3.32 to −2.99) (Supplementary Table S7; Supplementary Figure S3D). Additionally, the Syrian Arab Republic (EAPC = 5.28 [95% CI, 3.84 to 6.74]) had the fastest ASPR increase in terms of skull fracture (Supplementary Table S10; Supplementary Figure S3F).

**Figure 4:**
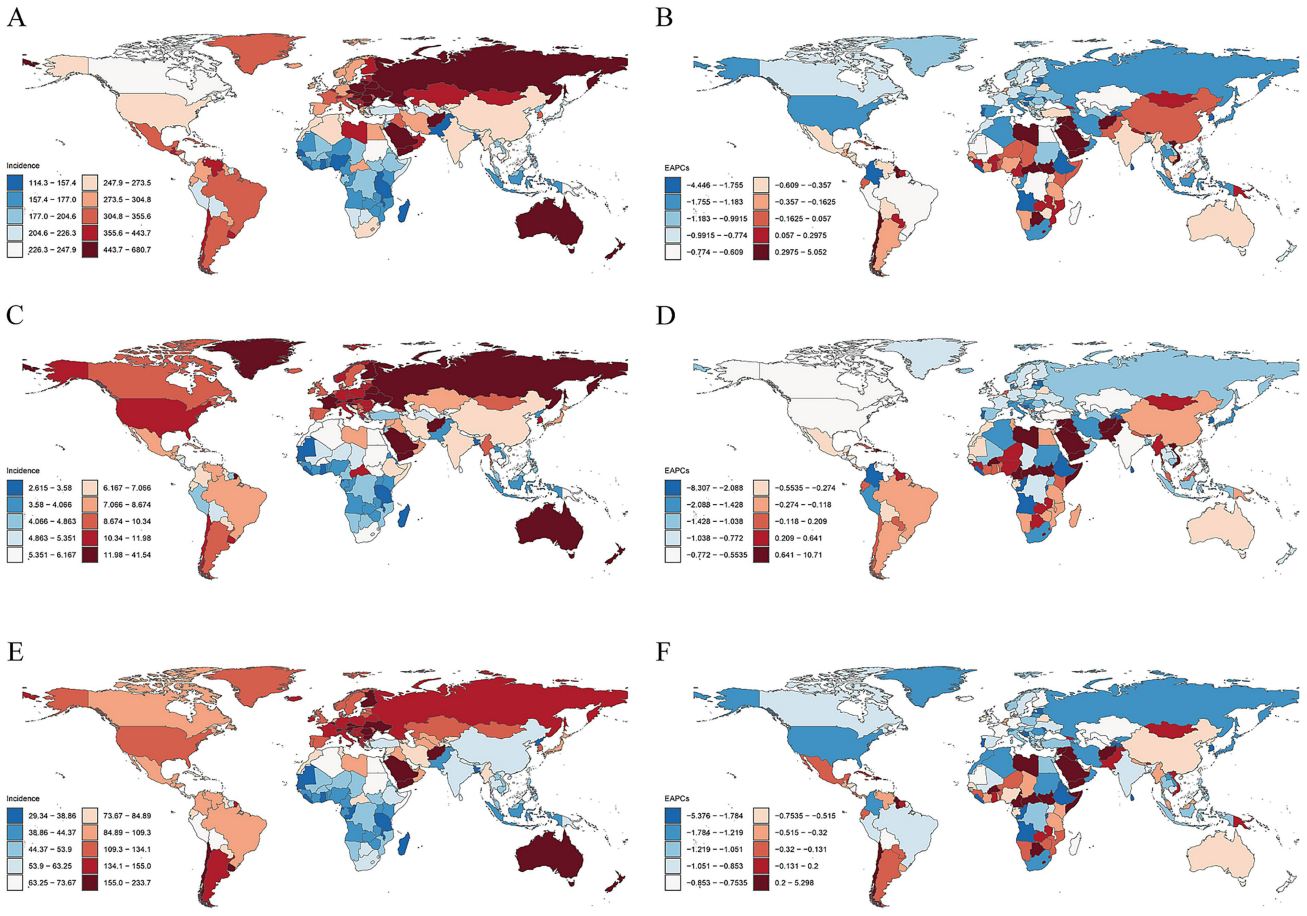
National ASIRs in 2021 and their EAPCs from 1990 to 2021 for TBI, SCI and skull fracture. ASIRs of TBI **(A)**, SCI **(C)** and skull fracture **(E)**. EAPCs in the ASIRs of TBI **(B)**, SCI **(D)** and skull fracture **(F)**. ASIR, age-standardized incidence rate; EAPC, estimated annual percentage change; TBI, traumatic brain injury; SCI, spinal cord injury.

In 2021, the ASIR of TBI across 204 countries or territories ranged from 114.3 to 680.68 per 100,000 people. The highest ASIR of TBI was reported in Saudi Arabia (680.68), Afghanistan (673.01), and Slovenia (621.57), whereas the lowest rate was reported in Bangladesh (114.3) (Supplementary Table S3; Figure 4A). Concurrently, Afghanistan (41.54) was estimated to have the highest ASIR attributed to SCI in 2021, followed closely by Yemen (22.46) and New Zealand (15.81), and the lowest was noted in Tonga (2.61) (Supplementary Table S6; Figure 4C). The highest ASIRs of skull fracture were observed in Afghanistan (233.7), New Zealand (232.97) and Australia (192.75). Conversely, the lowest ASIR was in Tonga (29.34) (Supplementary Table S9; Figure 4E). The largest change in the ASIR of TBI from 1990 to 2021 occurred in the Syrian Arab Republic, where the EAPC of the ASIR was 5.05 (95% CI: 2.93 to 7.22), and Burundi exhibited the most significant downward trend, at an EAPC of −4.44 (95% CI: −6.79 to −2.05) (Supplementary Table S3; Figure 4B). Notably, from 1990 to 2021, the Syrian Arab Republic also experienced the most significant upward trend in the ASIRs of SCI (EAPC = 10.71 [95% CI, 6.78 to 14.78]) and skull fracture (EAPC = 5.23 [95% CI, 3.18 to 7.46]) at the same time. In contrast, most regions exhibited downward trends, with Timor-Leste showing the most pronounced declines not only in the ASIR of SCI (EAPC = −8.31 [95% CI, −10.86 to −5.68]) but also in the ASIR of skull fracture (EAPC = −5.38 [95% CI, −6.94 to −3.79]) (Supplementary Tables S6, S9; Figures 4D,F).

The three countries with the highest ASYR of TBI in 2021 were Saudi Arabia (177.76), Slovenia (142.09) and Afghanistan (138.75), whereas Madagascar (25.84) had the lowest rate (Supplementary Table S5; Supplementary Figure S4A). The ASYR of SCI in 2021 was highest in Afghanistan (276.53), the Syrian Arab Republic (241.99) and Iraq (9493.2 (178.84)). Conversely, Malawi (20.82) had the lowest ASYR estimates (Supplementary Table S8; Supplementary Figure S4C). The ASYR of TBI in Portugal (EAPC = −2.51 [95% CI, −2.63 to −2.38]) showed the most significant downward trend from 1990 to 2021, and the Syrian Arab Republic (EAPC = 2.32 [95% CI, 1.66 to 2.98]) experienced the greatest increase in the ASYR (Supplementary Table S5; Supplementary Figure S4B). For SCI, the fastest increasing trend and declining trend in the ASYR were noted in Burundi (EAPC = 6.61 [95% CI, 4.3 to 8.97]) and Mozambique (EAPC = −3.47 [95% CI, −3.62 to −3.32]), respectively (Supplementary Table S8; Supplementary Figure S4D).

### Age and sex patterns

**Figure 5:**
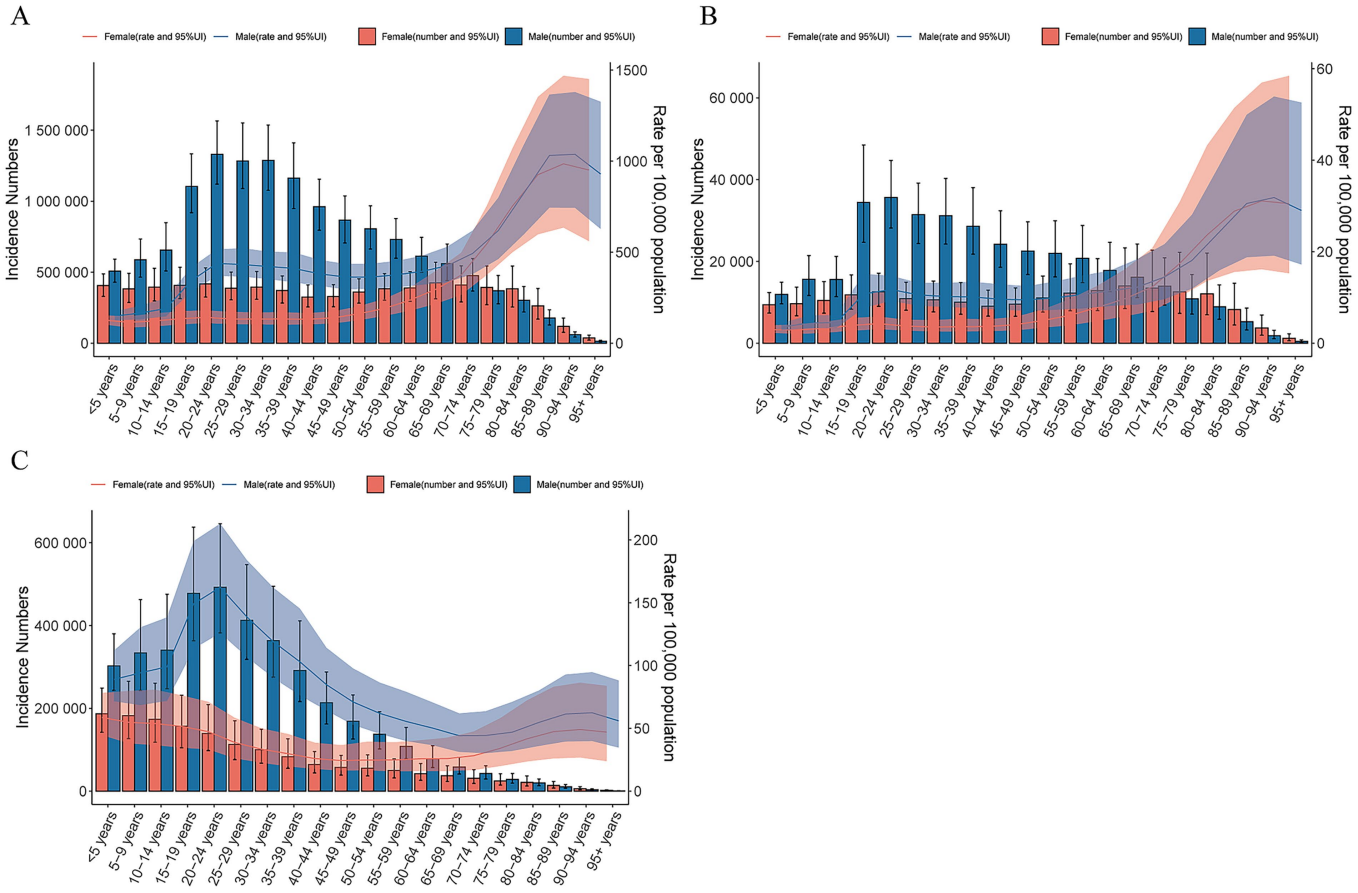
Global cases and age-standardized incidence rates of TBI **(A)**, SCI **(B)** and skull fracture **(C)** per 100,000 people by age and sex, 2021. Shading indicates the upper and lower limits of the 95% uncertainty intervals.

In 2021, the 20–24-year age group exhibited the highest number of incident TBI cases, followed by the 30–34-year age group. Similarly, the highest incidence rates of SCI and skull fractures were also observed in younger age groups. Globally, the incidence rate of TBI began to increase significantly in the 50–54-year age group in 2021, peaking in the 85–89-year age group. Notably, across all age groups, both incidence rates and case counts of TBI, SCI, and skull fractures were higher in men than in women. Additionally, YLD rates due to TBI and SCI were approximately sixfold higher in males than in females, indicating that the burden of these injuries (particularly TBI) was consistently greater in men than in women within the same age group (Figure 5; Supplementary Figures S6, S7).

### Burden from TBI, SCI and skull fracture attributable to leading risk factors

Notable disparities in the risk-attributable burdens of TBI, SCI, and skull fractures were observed across the 21 geographical regions. This study identified the leading risk factors associated with CNS injury burden in 2021 and elucidated the relative contributions of various risk factors to TBI, SCI, and skull fractures across global populations.

**Figure 6:**
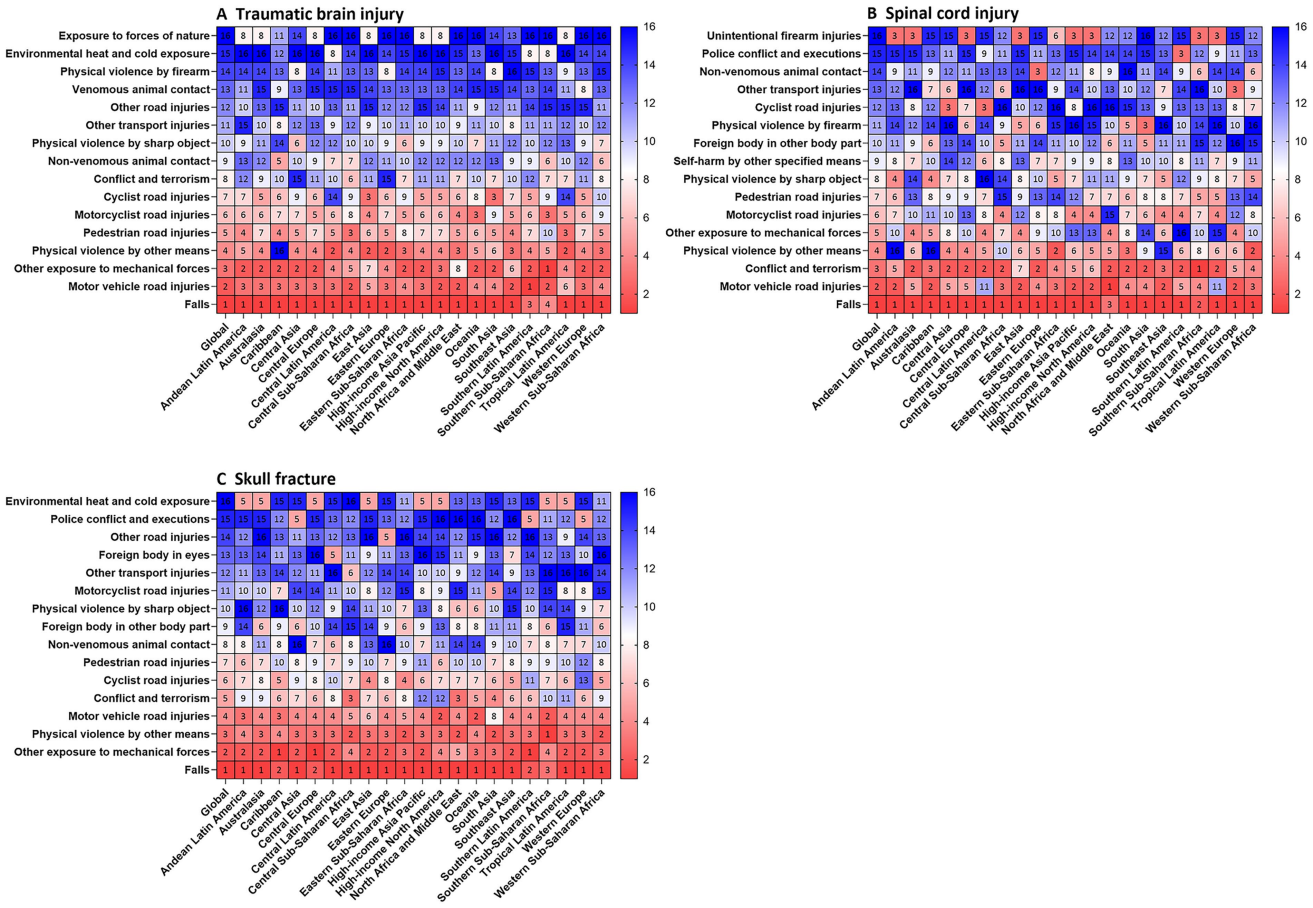
Ranked contribution of risk factors to the age-standardized incidence rates of TBI **(A)**, SCI **(B)** and skull fracture **(C)** by region, 2021. Risk factors are ranked from 1 (leading risk factor for age-standardized death; dark red) to 16 (lowest risk factor for age-standardized death; dark blue). TBI, traumatic brain injury; SCI, spinal cord injury.

In most regions, falls were the leading specific risk factor for TBI-related ASIR. However, in Southern Latin America, physical violence via other means was the primary specific risk factor, while pedestrian road injuries constituted the primary risk factor for TBI in Southern Sub-Saharan Africa (Figure 6A). Furthermore, falls remain a predominant risk factor for SCI globally, whereas conflict and terrorism constitute the top specific risk factors in North Africa and the Middle East. Additionally, firearm-related physical violence ranked as the second leading specific risk factor for SCI in Central Latin America and Tropical Latin America (Figure 6B). In East Asia, cyclist road injuries ranked as the third leading specific risk factor for TBI, while exposure to forces of nature was the fourth leading specific risk factor for TBI in the Caribbean. In Southeast Asia, police conflict and execution constituted the fourth leading specific risk factor for SCI. In Southern Sub-Saharan Africa, sharp object-related physical violence was the fifth leading specific risk factor for TBI, and in South Asia, contact with nonvenomous animals ranked as the fifth leading specific risk factor for SCI.

From a comprehensive perspective, although falls were the leading specific risk factor for global CNS injury burden in 2021, motor vehicle road injuries, motorcyclist road injuries, and pedestrian road injuries also emerged as important global risk factors (Figure 6A–C). Over 2000–2021, while annual trends in the risk factors for the three types of CNS injuries have fluctuated, they have remained generally stable on the whole (Supplementary Table S12; Supplementary Figure S11).

Stratifying the three CNS injury types by sex and age revealed distinct risk factor distributions for the same injury across groups. Females were more susceptible to traffic- and road safety-related injuries than males, whereas males with CNS injuries had higher proportions of two specific risk factors: other transport injuries and other mechanical force exposure. By age group (<20 years, 20–54 years, >55 years), younger (≤20 years) and older (>55 years) individuals were more prone to road injuries. In contrast, the middle-aged (20–54 years) had unique high-risk factors for CNS injuries, with significantly higher proportions of conflict/terrorism, sharp object-related violence, and firearm-related violence compared to adolescents and older adults (Supplementary Figure S8).

### Future forecasts of the global burden of encephalitis

From 2021 to 2040, the global burden of neurological injuries is projected to change significantly. The ASIR of TBI is expected to decline globally, dropping from ~65 to ~52 per 100,000 population (a ~ 20% reduction) over this period; incident TBI cases are projected to decrease from ~5.1 million to ~4.7 million (Supplementary Figures S9A, S10A). The ASIR of SCI is also forecast to decline globally, falling from ~7 to ~5 per 100,000, with SCI incident cases decreasing from ~550,000 to ~490,000 (Supplementary Figures S9B, S10B). For skull fractures, the global ASIR is anticipated to decrease from ~71 to ~50 per 100,000, while incident cases are projected to drop from ~5.5 million to ~4.5 million (Supplementary Figures S9C, S10C).

## Discussion

TBI, SCI, and skull fractures are three major types of CNS injuries and constitute a significant global health concern. In this study, we present the most recent global trends in CNS injury burden, provide comprehensive estimates of CNS injury incidence, prevalence, and YLDs, and analyze their temporal trends worldwide—including stratifications by region, age, and SDI. Besides, we predicted the developmental trends in the burden of TBI, SCI, and skull fracture till 2040. Although the total number of prevalent and incident cases increased moderately from 1990 to 2021, ASIR and ASPR declined—partly reflecting advances in CNS injury treatment and prevention. However, notable regional and national disparities persist.

Given that TBI and SCI are leading causes of death and long-term disability among adults, over half of all global TBI and SCI cases in 2021 occurred in adolescents and middle-aged populations, imposing a heavier social and economic burden than other injuries. With respect to the risk factors for CNS injuries, we conducted a comprehensive analysis of the temporal trends in the contribution ratios of specific factors across different age groups and gender groups. The latest data from GBD 2021 can assist policymakers in identifying recent risk factors and trends, facilitating the development of more targeted prevention and intervention strategies. This underscores the critical importance of analyzing the global burden, trends, and attributable risk factors of CNS injuries from a macro perspective.

The aforementioned risk factors for TBI, SCI, and skull fractures are increasingly prevalent and incident in populations across high- and high-middle-SDI regions, and correlate with high rates of CNS injuries in these areas (16). Previous studies have demonstrated that CNS injuries are significantly associated with various socioeconomic factors and lifestyles. Notably, rapid societal development toward a high socioeconomic status often results in population aging and an elevated incidence of trauma (17). In aging nations, falls at home, on roads, and in public spaces—amplified by frailty and environmental risks—drive high CNS injury mortality and morbidity among the older adults via heightened trauma severity and care delays. Falls, as the leading cause of SCI, TBI, and skull fractures, may be driven by factors such as impaired vision, frailty, alcohol abuse, insufficient protective awareness, and environmental factors; these risk factors tend to co-occur in countries with elevated ASIRs of CNS injuries (18). With population aging, YLDs have increased significantly in all populous countries over the past three decades. In some high-SDI countries, population aging has driven a rise in the incidence of degenerative neurological diseases (e.g., Parkinson’s disease, Progressive Supranuclear Palsy, Alzheimer’s disease, dementia with Lewy bodies) and acute stroke events (e.g., intracerebral hemorrhage, cerebral infarction), both of which constitute key risk factors for falls. Advances in diagnostic technologies have facilitated the detection of previously unrecognized mild or subclinical neurological impairments, expanding the pool of reported cases. Additionally, advances in treatments for neurological diseases have prolonged patient survival, expanding the population living with these conditions and increasing the burden of long-term care. Aging-wise, Germany’s 65 + population accounts for 45% of fall-induced CNS injuries, with 30% of cases untreated due to care gaps. Italy’s 70 + cohort sees 25% higher SCI from Alzheimer’s-related wandering. More details and source of data could be found in Supplementary material 3.

Beyond age-related structural factors, these CNS injuries are more likely to be secondary to rapid urbanization. Rapid urbanization and industrial development have led to a rising frequency of traffic accidents, which in turn has resulted in a persistently high burden of CNS injuries—particularly in Africa and South Asia. The number of traffic incidents is proportional to rising traffic volume. A study has concluded that traffic accidents remain the leading cause of premature death globally (19). Moreover, urbanization-related issues—including adverse and unsafe driving behaviors, expanding road networks, rising population density, and increased motor vehicle ownership—are closely associated with various road injuries and have intensified. National policies that overemphasize the speed of urbanization at the expense of public health support exacerbate the encroachment of high-density constructions on available road spaces, maldistribution of healthcare resources between urban and rural areas, neurodegenerative changes linked to pollution exposure, and mental health stress—thereby indirectly elevating the burden of neurological impairments associated with rapid urbanization. Rapid urbanization leads to a growing number of factories and workshops, rapid development of manufacturing, and consequently more CNS injuries caused by industrial machinery. Greater YLDs of SCI, TBI, and skull fractures observed in high- and high-middle-SDI regions are related to this. Among European countries, urbanization and aging drive burdens. As noted in WHO and World Bank reports, Germany’s Berlin has 40% of e-scooter accidents causing TBI, due to dense traffic and young riders’ risk-taking. Italy’s Milan records 15% higher neurodegenerative-related falls in polluted zones. More details and source of data could be found in Supplementary material 3. Thus, it is critical for all nations—particularly those with higher SDI—to address the challenges posed by population aging and the adverse impacts stemming from rapid urbanization.

Countries with low SDI face a disproportionately high burden of TBI and SCI, closely linked to lifestyle and behavioral patterns. Motorcycle-dominated transport, often without helmets, combined with prevalent alcohol-impaired driving, elevates crash-related trauma risks. In informal sectors like construction, inadequate safety gear and unregulated practices increase falls and crush injuries. Substandard housing and poor urban infrastructure raise household fall risks, particularly among children and the older adults. Additionally, delayed post-injury care due to limited emergency services exacerbates neurological damage, amplifying the overall burden. Low SDI countries face rising CNS injury burdens due to inadequate healthcare, unhealthy lifestyles, and lax traffic regulations. According to the overview released by the World Health Organization and the World Bank Report, take India and Nigeria: data shows India has <1 neurosurgeon per 100,000 people, with 60% of rural trauma centers lacking CT scanners, delaying TBI/SCI management. Nigeria’s rural areas have 90% of primary clinics without spinal immobilization tools, increasing secondary injuries. Lifestyle-wise, India’s urbanization has raised motorcycle usage by 40% since 2010, with 75% of riders unhelmeted, boosting skull fractures.

Lax traffic legislation and inadequate enforcement reduce deterrence, perpetuating risky behaviors like helmet non-compliance, speeding, overloading, wrong-way driving, distracted or reckless driving, impaired driving and unregistered vehicle operation. These practices amplify crash severity, directly correlating with elevated neurological injury mortality and morbidity, serving as critical drivers of their high burdens. Traffic laws in both are poorly enforced: Nigeria’s road fatality rate (35/100,000) is 8x that of high-income countries, while India’s rural areas see 70% of motorists ignoring speed limits, fueling crash-related CNS injuries. More details and source of data could be found in Supplementary material 3.

According to the latest findings from the International Initiative for Traumatic Brain Injury Research, TBI is a leading cause of trauma-related mortality in middle-aged populations (30–59 years) and ranks as the most lethal cause of CNS injury (20). This is primarily due to multimodal management of TBI patients often necessitates surgical interventions, including decompressive and intracranial pressure monitoring. Notably, the high-income Asia-Pacific region exhibited the lowest a ASYR for TBI, linked to the relatively high ASIR in this region in 2021, which facilitated the implementation of effective treatment strategies and enhanced acute trauma care—both of which significantly alleviated the burden of TBI. These regions typically feature more sophisticated systems for early surgical intervention (e.g., intracranial hematoma evacuation), along with rational medication use in the acute phase (e.g., mannitol, methylprednisolone), and greater accessibility to post-traumatic seizure prophylaxis and agitation management (21, 22). Limited access to quality healthcare—including neurosurgical intensive care units and rehabilitation facilities—may exacerbate the burden of TBI in resource-constrained settings.

For traumatic SCI, the therapeutic priority lies in preventing and mitigating secondary injuries, particularly the prevention of spinal shock and spinal edema. Early recognition of SCI in prehospital settings is critical to avoiding secondary spinal cord damage during transportation. However, a lack of relevant medical knowledge contributes to the high burden of SCI in certain regions and nations (23). Similarly, intervention strategies encompassing immediate spinal immobilization, critical care, timely spinal surgery for neural element decompression, and meticulous care for complications prevention have been implemented in large-scale emergency trauma center in high-income Asia-Pacific regions, which can significantly alleviate the burden of SCI. Notably, healthcare resources and services vary across regions and SDI quintiles, underscoring the necessity for regions and nations to develop context-specific strategies to guide clinical practice and extend benefits to a broader global population—particularly in low- and middle-income countries.

In the context of significant advancements in policy reforms spearheaded by the WHO—focused on enhancing treatment quality, addressing population aging, optimizing healthcare resources distribution, strengthening road safety regulations and promoting road safety education—global ASYR and ASIR for central nervous system CNS injuries have declined over the past three decades, with regional disparities mitigated to some extent. However, substantial improvements remain necessary: the persistently rising burden underscores that current disease prevention efforts are markedly insufficient in regions identified as high-risk. Our analysis revealed a notable increase in CNS injury burden in specific countries, including Syria, Burundi, Afghanistan, Iraq, Yemen, and Ukraine. This phenomenon merits attention, and we offer preliminary insights: the ongoing conflict in Syria likely constitutes a key risk factor for the marked rise in TBI and SCI ASIRs. This increase is primarily attributable to firearm-related violence, conflict, terrorism, and other forms of physical violence, alongside risk factor transitions in these regions—characterized by a shift from constraints in healthcare systems to a growing prevalence of threats to personal safety. A similar pattern of elevated ASPR in war-affected contexts is observable in other nations, such as those in the Middle East enduring regional conflicts, where residents face a multi-fold risk of CNS injury. It has been suggested that war potentially exerts substantial adverse impacts on a country’s healthcare system and impairs access to medical and surgical services (24, 25). Victims of conflict- and terrorism-related traumatic injuries may lack access to appropriate surgical and medical interventions—care that would help alleviate disability severity. Nevertheless, severe TBI and SCI are life-threatening for individuals in war-torn nations, as these populations frequently experience elevated early and delayed mortality rates, alongside functional impairments, due to missing optimal treatment time and medical resource limitations. Undoubtedly, prevention remains the most effective strategy to reduce the burden of such CNS injuries, underscoring the need for international collaboration to mitigate the rising incidence of traumatic CNS injuries in war-affected regions.

Furthermore, against the backdrop of a global decline in the ASPR, ASIR, and ASYR for three types of CNS injuries between 1990 and 2021, TBI and SCI impose a heavier health burden on populations in the Caribbean and Oceania—regions where ASIR, ASYR, and EAPC have risen. Exposure to natural hazards, such as typhoons and tsunamis, may represent a primary driver of the higher-than-expected burden of CNS injuries (26). Strengthening policies for managing traumatic CNS injuries and prevention strategies should incorporate current population dynamics and healthcare practices. For instance, efforts in these regions include humanitarian relief initiatives, monitoring of extreme weather events, and early recovery interventions. In contrast, the declining trends in ASIR and ASYR in high-income Asia-Pacific and high-income North American regions reflect a substantial reduction in the burden of CNS injuries. These gains can be attributed to the contributions of social development, technological advances, industrialization, and improvements in medical resources (27, 28). These findings underscore the need for regions and nations to tailor their prevention and treatment strategies not only to internationally recognized guidelines for the management of CNS injuries but also to local contexts, while prioritizing regional risk factors.

Our analyses in this study also examined sex differences in the disease burden of TBI, SCI, and skull fractures. Notably, we observed an uneven sex distribution in the incidence of CNS injuries: young-to-middle-aged men were consistently more likely to be affected by TBI, SCI, and skull fractures than women. This finding might be due to societal and cultural factors in which men are more likely to be exposed to road injuries, injuries linked to social role stress, armed conflicts and alcohol exposure (29). From a societal role perspective, men are disproportionately engaged in high-risk occupations (e.g., construction, mining, heavy transportation) and activities (e.g., motorcycle riding, competitive contact sports) due to gendered labor divisions and social expectations, these factors may contribute to the rising disease burden of TBI, SCI, and skull fractures among males aged 15–39 years. Females face higher traffic-related CNS injury risk due to greater pedestrian exposure and lower protective gear use.

From a temporal trend standpoint, the novel coronavirus (COVID-19) pandemic—emerging in late 2019 and early 2020—impacted the implementation of prevention and treatment measures for TBI, SCI, and skull fractures. These impacts stemmed from the reallocation of medical resources and substantial shifts in social and productive activities due to COVID-19. Furthermore, inflection points in the overall temporal trend of CNS injury burden across specific regions and SDI quintiles were associated with extraordinary traumatic events, including wars and natural disasters (30, 31). From 2000 to 2021, the annual trends of risk factors for the three types of CNS injuries exhibited fluctuations but remained generally stable. We categorized all risk factors into five groups: Falls, Transport-related injuries, Non-traffic mechanical injuries, Interpersonal Violence, and Conflict and Terrorism. This stability may stem either from the persistence of unaddressed risk factors or from improvements in one subcategory being offset by the emergence of new risk factors amid societal development over time. While the risk factors for these three CNS injuries display certain macro-level differences, aging-related falls, as well as mechanical and traffic-related injuries associated with rapid urbanization, constitute three pressing issues that demand immediate attention—consistent with our earlier discussion.

The primary strength of this study is its comprehensive characterization of the burden of TBI, SCI, and skull fractures, utilizing the latest national estimates from the GBD 2021 study. However, our study has several inherent limitations. First, while our data were derived from an authoritative disease burden database, they may not fully capture the epidemiological profiles of these injuries. Additionally, data accuracy and consistency vary across regions, potentially introducing biases or inaccuracies. Data scarcity, a longstanding challenge in GBD studies, hinders the generation of more precise estimates. Second, our analysis focused exclusively on three traumatic CNS injuries: TBI, SCI, and skull fractures. Third, the study’s temporal scope (1990 onward) may be influenced by evolving diagnostic criteria and medical technologies over time. Thus, while our findings offer valuable insights into global CNS injury trends, they should be interpreted cautiously. Finally, TBI and SCI are subtyped by trauma severity or level (e.g., mild TBI, moderate/severe TBI, cervical SCI, subcervical SCI)—critical parameters that warrant inclusion. Future large-scale global epidemiological studies are therefore needed to expand the analysis of CNS injury burden and deepen understanding of traumatic injuries.

## Conclusion

In summary, this study characterized the global burden and risk factors of TBI, SCI, and skull fractures using GBD 2021 data, and analyzed trends from 1990 to 2021 at the global, regional, and national levels. Despite a slight downward trend in the global burden of these injuries over this period, CNS injuries remain a pressing public health challenge, with persistent regional disparities. To address the limitations of GBD studies, we advocate developing and implementing more advanced analytical methods to expand and validate disease burden estimates. This study provides a theoretical foundation for designing more effective prevention strategies and optimizing the allocation of limited resources. Tailored interventions are critical to alleviating the global burden of CNS injuries and advancing health equity.

## Contributions to the literature

We assessed the global burden of central nervous system (CNS) from 1990 to 2021, providing new and robust evidence of traumatic brain injury (TBI), spinal cord injury (SCI), and skull fracture burden worldwide. By using vital statistics and hospital admission data for more than 100 million individuals, we have provided more detailed and evidence-based estimates of the cause and risk factors of TBI, SCI and skull fracture than have been available previously. The analysis is stratified by sex, age, and SDI at global, regional, and national levels, allowing for a nuanced understanding of how CNS injury affects different populations.

## Data Availability

Publicly available datasets were analyzed in this study. This data can be found at: https://vizhub.healthdata.org/gbd-results/.

## Footnotes

^1^ https://vizhub.healthdata.org/gbd-results/

## Supplementary material

SUPPLEMENTARY FIGURE S1 ASPR in 2021 and their annual changes every decade from 1990 to 2021 for TBI, globally and by 21 GBD regions **(A)**, and EAPCs in the ASPR across 21 GBD regions, 1990–2021 **(B)**. ASPRs in 2021 and their annual changes every decade from 1990 to 2021 for SCI, globally and by 21 GBD regions **(C)**, and the EAPCs in the ASPR across 21 GBD regions, 1990–2021 **(D)**. ASPRs in 2021 and their annual changes every decade from 1990 to 2021 for skull fracture, globally and by 21 GBD regions **(E)**, and EAPCs in the ASPR across 21 GBD regions, 1990–2021 **(F)**. ASPR, age-standardized prevalence rate; EAPC, estimated annual percentage change; TBI, traumatic brain injury; SCI, spinal cord injury.

SUPPLEMENTARY FIGURE S2 ASYRs in 2021 and their annual changes every decade from 1990 to 2021 for TBI, globally and by 21 GBD regions **(A)**, and the EAPCs in the ASYR across 21 GBD regions from 1990–2021 **(B)**. ASPRs in 2021 and their annual changes every decade from 1990 to 2021 for SCI, globally and by 21 GBD regions **(C)**, and EAPCs in the ASYR across 21 GBD regions, 1990–2021 **(D)**. ASYRs in 2021 and their annual changes every decade from 1990 to 2021 for skull fracture, globally and by 21 GBD regions **(E)**, and EAPCs in the ASYR across 21 GBD regions, 1990–2021 **(F)**. ASYR, age-standardized years lived with disability rate; EAPC, estimated annual percentage change; TBI, traumatic brain injury; SCI, spinal cord injury.

SUPPLEMENTARY FIGURE S3 National ASPR in 2021 and the EAPCs from 1990 to 2021 for TBI, SCI and skull fracture. ASPRs of TBI **(A)**, SCI **(C)** and skull fracture **(E)**. EAPCs in the ASPRs for TBI **(B)**, SCI **(D)** and skull fracture **(F)**. ASPR, age-standardized prevalence rate; EAPC, estimated annual percentage change; TBI, traumatic brain injury; SCI, spinal cord injury.

SUPPLEMENTARY FIGURE S4 National ASYR in 2021 and their EAPCs from 1990 to 2021 for TBI, SCI and skull fracture. ASYRs of the TBI **(A)**, SCI **(C)** and skull fracture **(E)**. EAPCs in the ASYRs for TBI **(B)**, SCI **(D)** and skull fracture **(F)**. ASYR, age-standardized years lived with disability rate; EAPC, estimated annual percentage change; TBI, traumatic brain injury; SCI, spinal cord injury.

SUPPLEMENTARY FIGURE S5 Temporal trend of CNS injury burden globally across 204 countries and territories. **(A)** Percentage change in the incidence of TBI across 204 countries in 1990 and 2021. **(B)** Percentage change in the incidence of SCI across 204 countries in 1990 and 2021. **(C)** Percentage change in the incidence of skull fracture across 204 countries in 1990 and 2021. TBI, traumatic brain injury; SCI, spinal cord injury.

SUPPLEMENTARY FIGURE S6 Global cases and age-standardized incidence rates of TBI **(A)**, SCI **(B)** and skull fracture **(C)** per 100,000 people by age and sex, 2021. Shading indicates the upper and lower limits of the 95% uncertainty intervals.

SUPPLEMENTARY FIGURE S7 Global cases and age-standardized years associated with the disability rates of TBI **(A)**, SCI **(B)** and skull fracture **(C)** per 100,000 people by age and sex in 2021. Shading indicates the upper and lower limits of the 95% uncertainty intervals.

SUPPLEMENTARY FIGURE S8 Ranked contribution of risk factors to the age-standardized incidence rates of TBI **(A)**, SCI **(B)** and skull fracture **(C)** grouped by gender and age group, 2021. Risk factors are ranked from 1 (leading risk factor for age-standardized death; dark red) to 16 (lowest risk factor for age-standardized death; dark blue). TBI, traumatic brain injury; SCI, spinal cord injury.

SUPPLEMENTARY FIGURE S9 Future forecasts of the global burden of CNS injury. **(A)** ASIR of TBI. **(B)** ASIR of SCI. **(C)** ASIR of skull fracture. ASIR, age-standardized incidence rates; TBI, traumatic brain injury; SCI, spinal cord injury.

SUPPLEMENTARY FIGURE S10 Future forecasts of the global burden of CNS injury. **(A)** Age-standardized incidence cases of TBI. **(B)** Age-standardized incidence cases of SCI. **(C)** Age-standardized incidence cases of skull fracture. TBI, traumatic brain injury; SCI, spinal cord injury.

SUPPLEMENTARY FIGURE S11 The annual change trend of the contribution proportion of the five types of risk factors from 2000 to 2021. **(A)** TBI. **(B)** SCI. **(C)** Skull fracture. TBI, traumatic brain injury; SCI, spinal cord injury.
