# Supplementary material for: Global, regional, and national burdens of traumatic brain injury, spinal cord injury, and skull fracture and their attributable risk factors from 1990 to 2021: a systematic analysis of the global burden of disease study 2021
Source: Front Public Health. 2025 Aug 20;13:1622693. doi: 10.3389/fpubh.2025.1622693 (PMC12405261; doi:10.3389/fpubh.2025.1622693)
Supplement: Supplementary file 1 [file Presentation_1.pdf]

In GBD database research, model-based imputation methods aim to estimate missing data through statistical modeling, thereby enhancing data completeness and analytical accuracy. Common approaches and their principles are as follows:

Expectation-Maximization (EM) algorithm: It iteratively updates estimates of missing values. Initially, it makes initial guesses about missing values based on existing observed data. Then, these guesses and observed data are used to estimate model parameters, which in turn update the missing value estimates. This process repeats until convergence, yielding the imputed results for missing values.

Multiple Imputation (MI): Instead of filling missing values with a single value, it generates multiple plausible imputed values to create several complete datasets. Each imputed dataset is analyzed separately, and the results are combined to derive the final conclusions. This method better accounts for the uncertainty of missing values.

Multivariate Imputation by Chained Equations (MICE): For datasets with multiple variable types (continuous, categorical, etc.), it builds a regression or classification model for each variable with missing values, using other variables as predictors to impute the missing values. Through iterative cycles, missing values of each variable are imputed sequentially until all are reasonably filled.

Spatio-Temporal Gaussian Process Regression (ST-GPR): Frequently used in GBD studies to generate complete retrospective time series, it incorporates temporal and spatial characteristics as variables. By modeling correlations between data points via Gaussian processes, it predicts missing values at unobserved spatio-temporal locations using known data, effectively capturing temporal and spatial trends in disease burden data, suitable for imputing missing disease-related data across global regions.

Geospatial covariate-based modeling: It constructs models using geospatial covariates (e.g., per capita income, smoking rates). Taking the geographic location of missing data and related covariates as inputs, the model predicts disease burden values for that

location. For example, spatial interpolation estimates disease burden in low-income regions, leveraging spatial data distribution features for imputation.
