# Supplementary material for: Global, regional, and national burdens of traumatic brain injury, spinal cord injury, and skull fracture and their attributable risk factors from 1990 to 2021: a systematic analysis of the global burden of disease study 2021
Source: Front Public Health. 2025 Aug 20;13:1622693. doi: 10.3389/fpubh.2025.1622693 (PMC12405261; doi:10.3389/fpubh.2025.1622693)
Supplement: Supplementary file 2 [file Presentation_2.pdf]

**BAPC Models for Projections**

The Bayesian Age-Period-Cohort (BAPC) model is a robust statistical framework integrating age, period, and cohort effects to analyze temporal trends in health outcomes. It employs Bayesian inference with integrated nested Laplace approximations (INLA) for efficient computation, quantifying uncertainties in parameter estimates. Widely used in epidemiological studies, it enables trend projection by leveraging historical data, aiding in understanding disease dynamics and informing public health strategies[1]. Analysis Tools Help Page is (https://folk.ntnu.no/andrerie/software.html)

Key features of BAPC models include:

1. Generation of age-specific and age-standardized projected rates.

2. Automatic addition of Poisson noise when interest lies in the predictive distribution.

## Reference

[1] de Lemos L, Guerra J A, Santos M, et al. **The Assessment for Disinvestment of Intramuscular Interferon Beta for Relapsing-Remitting Multiple Sclerosis in Brazil**[J]. *Pharmacoeconomics* 2018,36(2):161-173.
