# Supplementary material for: Global, regional, and national burdens of traumatic brain injury, spinal cord injury, and skull fracture and their attributable risk factors from 1990 to 2021: a systematic analysis of the global burden of disease study 2021
Source: Front Public Health. 2025 Aug 20;13:1622693. doi: 10.3389/fpubh.2025.1622693 (PMC12405261; doi:10.3389/fpubh.2025.1622693)
Supplement: Supplementary file 3 [file Presentation_3.pdf]

I. Data related to low SDI countries

Neurosurgeon density in India

Data source: WHO Global Health Workforce Statistics Database (to be retrieved via the GHO platform)

Access link: https://www.who.int/data/gho

Search keywords: "Neurosurgeons per 100,000 population India"

Note: The conclusion that India's neurosurgeon density is less than 1 per 100,000 population can be queried through this database, with data integrated from health department reports of various countries.

Equipment in primary healthcare facilities in Nigeria

Data source: World Bank's Nigeria Health System Assessment Report

Access path: Nigeria Overview: Development news, research, data | World Bank

Search keywords: "Primary healthcare facility equipment Nigeria"

Note: The World Bank's 2022 report indicates that 90% of primary healthcare facilities in rural Nigeria lack spinal immobilization tools. Relevant assessment documents can be obtained through the "Publications" section on its country page.

Motorcycle helmet usage rate in India

Data source: WHO's Global Status Report on Road Safety 2023

Direct link: https://www.who.int/publications/i/item/9789240073430

Chapter reference: Regional data on helmet usage in Chapter 3 "Risk factors and countermeasures".

Road traffic mortality rate in Nigeria

Data source: WHO Global Road Safety Database

Access link: https://www.who.int/data/gho/data/themes/topics/road-safety

Search keywords: "Road traffic mortality rate Nigeria"

Note: The road traffic mortality rate of 35 per 100,000 in Nigeria can be queried through this database, compared with the average level of high-income countries at approximately 4.3 per 100,000.

II. Data related to developed countries

Proportion of TBI in e-scooter accidents in Germany

Data source: Urban Traffic Injury Surveillance Report by WHO Regional Office for Europe

Access path:

https://link.wtturl.cn/?target=https%3A%2F%2Fwww.euro.who.int%2F&scene=im&aid=497858&lang=zh

Search keywords: "E-scooter accidents TBI Berlin"

Note: The conclusion that 40% of e-scooter accidents in Berlin result in TBI can be queried through the "Accidents and injuries" subcategory under the "Health topics" section of this regional office.

Association between pollution and falls in Italy

Data source: World Bank's Italy Environmental and Health Impact Assessment

Access path:

https://link.wtturl.cn/?target=https%3A%2F%2Fwww.worldbank.org%2Fen%2Fcountry%2Fitaly%2Fpublications&scene=im&aid=497858&lang=zh

Search keywords: "Air pollution and falls Milan"

Note: Data showing that the neurodegenerative fall rate in polluted areas of Milan is 15% higher can be obtained through the "Environment & Natural Resources" category on this page.

Aging and fall burden in Germany

Data source: WHO's Ageing and Health in Europe Report

Direct link:

https://link.wtturl.cn/?target=https%3A%2F%2Fwww.euro.who.int%2F__data%2Fassets%2Fpdf_file%2F0003%2F514500%2FAgeing-and-health-in-Europe-2023.pdf&scene=im&aid=497858&lang=zh

Chapter reference: Data on falls among people aged 65 and above in Germany in Chapter 4 "Falls and fractures".

SCI related to Alzheimer's disease in Italy

Data source: World Bank's Analysis of Healthcare Burden in Italy's Ageing Society

Access path: Italy Overview: Development news, research, data | World Bank

Search keywords: "Alzheimer's disease and spinal cord injury Italy"

Note: Data indicating that the incidence of SCI due to wandering among people aged 70 and above with Alzheimer's disease in Italy is 25% higher can be obtained through the "Publications" section on its country page.
